# The human hypothalamus coordinates switching between different survival actions

**DOI:** 10.1371/journal.pbio.3002624

**Published:** 2024-06-28

**Authors:** Jaejoong Kim, Sarah M. Tashjian, Dean Mobbs

**Affiliations:** 1 Department of Humanities and Social Sciences and Computation, California Institute of Technology, Pasadena, California, United States of America; 2 Neural Systems Program at the California, California Institute of Technology, Pasadena, California, United States of America; Universitat Jaume 1, SPAIN

## Abstract

Comparative research suggests that the hypothalamus is critical in switching between survival behaviors, yet it is unclear if this is the case in humans. Here, we investigate the role of the human hypothalamus in survival switching by introducing a paradigm where volunteers switch between hunting and escape in response to encounters with a virtual predator or prey. Given the small size and low tissue contrast of the hypothalamus, we used deep learning-based segmentation to identify the individual-specific hypothalamus and its subnuclei as well as an imaging sequence optimized for hypothalamic signal acquisition. Across 2 experiments, we employed computational models with identical structures to explain internal movement generation processes associated with hunting and escaping. Despite the shared structure, the models exhibited significantly different parameter values where escaping or hunting were accurately decodable just by computing the parameters of internal movement generation processes. In experiment 2, multi-voxel pattern analyses (MVPA) showed that the hypothalamus, hippocampus, and periaqueductal gray encode switching of survival behaviors while not encoding simple motor switching outside of the survival context. Furthermore, multi-voxel connectivity analyses revealed a network including the hypothalamus as encoding survival switching and how the hypothalamus is connected to other regions in this network. Finally, model-based fMRI analyses showed that a strong hypothalamic multi-voxel pattern of switching is predictive of optimal behavioral coordination after switching, especially when this signal was synchronized with the multi-voxel pattern of switching in the amygdala. Our study is the first to identify the role of the human hypothalamus in switching between survival behaviors and action organization after switching.

## Abstract 2

Studies in animal models suggest that the hypothalamus is critical for modulating the switch between survival behaviors. In this study, neuroimaging combined with computational modeling shows that the hypothalamus also modulates switching between survival actions in humans.

## Introduction

A vital determinant of an organism’s longevity is its ability to efficiently select and switch among survival states. This suggests that it would be highly advantageous to evolve a specialized neural circuit that coordinates the transient switching between survival states (e.g., hunting versus escape). The hypothalamus, a region involved in many of life’s basic functions, is one candidate region that is perfectly situated for such purposes. This conserved structure, present across all vertebrate species, has also been linked to many survival behaviors including escape, aggression, and hunting behaviors [1]. While the hypothalamus controls these basic survival states, it is also a key player in a larger network of brain regions that are involved in interoceptive states, defensive motor output, and learning and memory [2–4]. Given the role of the hypothalamus in self-preserving behaviors in mammals, one key question remains: Does the hypothalamus perform the same function in humans?

Mammalian studies have emphasized the role of the amygdala, periaqueductal gray (PAG), and prefrontal cortex in threat detection. These regions, however, seem to focus on the salience and scalability of the threat [5–9] rather than determining which survival action to pursue. Experiments on rodents have discovered that the hypothalamus controls both flight and freeze [10,11]. More recently, optogenetic and calcium imaging studies show that the ventromedial hypothalamus elicits the switch between aggression and mounting [12]. Furthermore, the lateral hypothalamus can induce switching between escaping and hunting behavior [13,14]. These survival behaviors, however, reflect the link between the most basic of motor reactions and cognitive heuristics that involve goal-directed survival behavior such as avoidance of future danger [6] and predictive hunting behavior [15,16]. Further, the hypothalamus is connected to both the frontal cortex and PAG [17], making it an intriguing interface between cognitive and reactive strategizing.

One reason for the lack of research on the human hypothalamus is that the hypothalamus contains several small nuclei that are below the resolution of fMRI; consequently, there is no experimental human analog for investigating this region [18]. To overcome these concerns, we take several approaches: (i) individual-specific segmentation of the hypothalamus was utilized to define hypothalamic ROIs by using a recent deep-learning-based convolutional neural network (CNN) algorithm [19]; (ii) since the hypothalamic signal is influenced by cerebral spinal fluid (CSF) pulsatility of the ventricle, we regressed out CSF signal [20]. (iii) We used 2-mm isotropic voxels, which have a better signal-to-noise ratio than smaller (1 to 1.5 mm) voxel imaging [21]; and (iv) we used multi-voxel pattern analyses (MVPA) to investigate the changing patterns of voxels in the hypothalamus when switching between survival behaviors. Compared to univariate analyses, MVPA is known to have a higher sensitivity to detect neural information by utilizing distributed multi-voxel patterns rather than using mean activation [22]. We also extended our multi-voxel approach to investigate network-level encoding of switching between survival behaviors by testing multi-voxel pattern synchronization between the hypothalamus and other regions using the informational connectivity (IC) [23–25].

**Fig 1:**
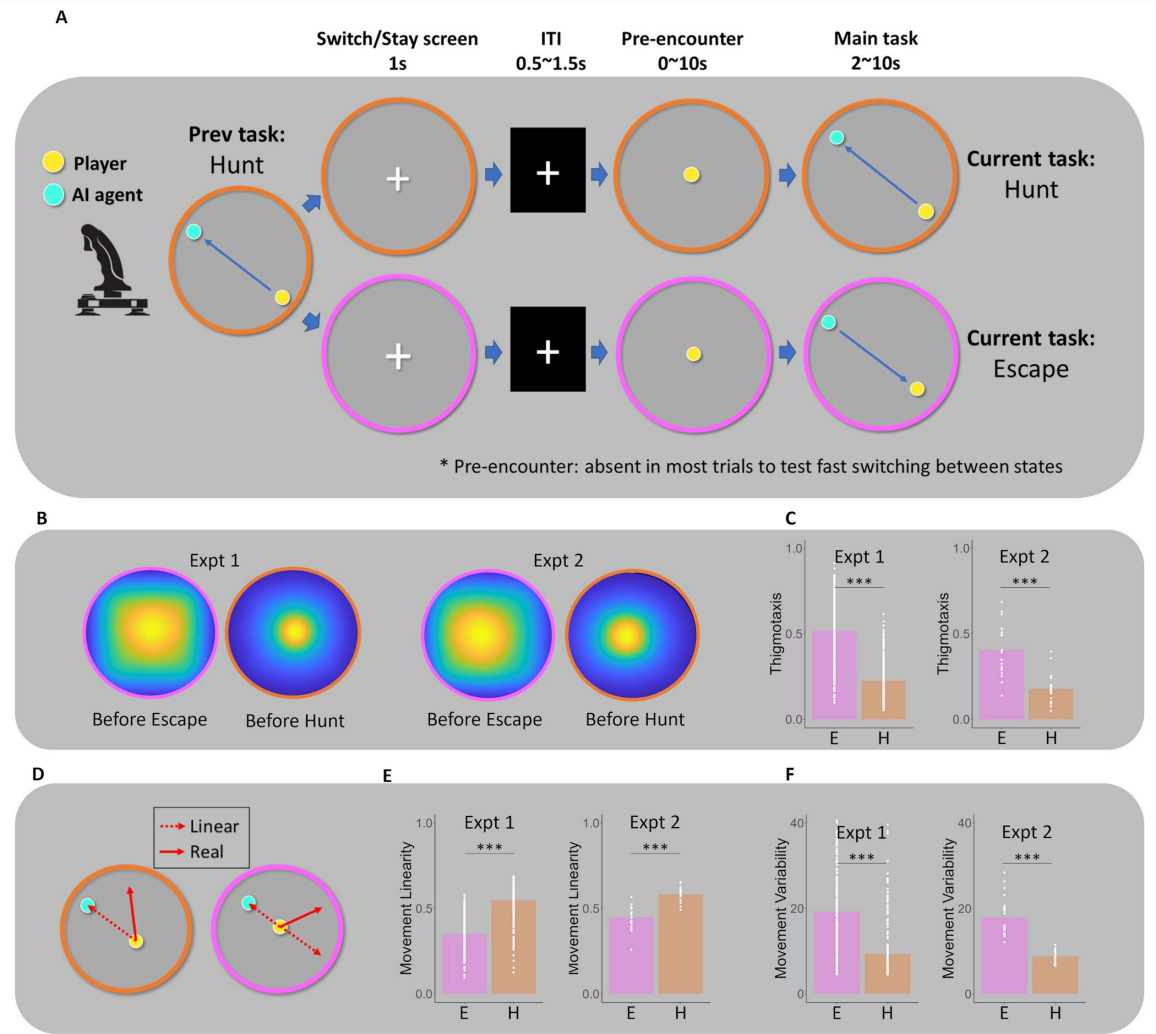
Experimental task and behavioral results. (A) Experimental paradigm; participants controlled an avatar (player; yellow circle) and either hunted (virtual prey) or escaped (virtual predator) from the cyan circle. At the start of every trial, participants are shown a 2D virtual arena with either a pink (in this example, signaling “Hunt” condition) or orange (in this example, signaling “Escape” condition) boundary for 1 s. In the “Hunt condition,” the participants should chase and attempt to capture a virtual prey. Conversely, in the “Escape condition,” the participants should escape from the virtual predator. Before starting the experimental task, participants occasionally entered a pre-hunt or pre-escape condition where participants prepared for the predator or prey before they entered the arena, allowing us to examine preparatory defensive or predatory movements. Players used an MRI-compatible joystick to complete all tasks. A successful hunt was defined as the capture of the prey before the termination of each trial. Successful escape was defined as not being captured. (B) Visualization of the visiting frequency in the pre-hunt and pre-escape period (yellow: high visiting frequency; blue low visiting frequency; normalized with a maximum visiting frequency). Players tend to move closer to the boundary (thigmotaxis) before escaping compared to hunting in both online and fMRI studies since both predator and prey tend to appear close to the center of the field. (C) Bar graphs show significant differences in thigmotaxis (a proportion of the time spent closer to the boundary; ranged between 0 and 1; ideally, the thigmotaxis would be close to 1 before the escape while it would be close to 0 before the hunt considering the expected location of the predator/prey appearance) before Escape compared to Hunt Conditions. (D, E) Movement difference between Hunt and Escape conditions. Linearity of the movement was defined as the movement that minimizes distance from the current position of the prey in the Hunt task and the movement that maximizes distance from the current position of the predator in the Escape task, which ranged between −1 and 1 where 1 means the movement toward ideal direction that minimize (maximize) distance from the current position of the prey (predator) and −1 means the movement toward the opposite direction of an ideal direction. (D) The movement was significantly more linear in the Hunt task than in the Escape task (Fig 1E; left-online study; right-fMRI study). (F) Movement variability in the Hunt and Escape task. Movement variability was defined as an average number of changes in direction per unit time (second; ranged between 0 and 59). Movement variability was significantly higher in the Escape task than in the Hunt task. ***p < 0.001, **p < 0.01, *p < 0.05. The data underlying this figure is available from https://osf.io/k5p3m/.

We developed an experimental paradigm that could examine the role of the hypothalamus in switching between hunting and escape in humans (Fig 1A). Using a virtual arena that mimics experimental environments used in rodents, subjects were asked to either hunt (virtual prey; cyan circle in Fig 1A) or escape from a computer agent (virtual predator). Participants also performed a control task that similarly involves switching between continuous motor movement as in the experimental task and does not involve survival behaviors such as escaping or hunting. This was done to test whether the hypothalamus is generally involved in switching between continuous movement or it is more specific to switching between survival behaviors such as escaping and hunting. Further, our paradigm allowed us to measure movements on 2 fronts: to determine if anxiety-like behaviors (i.e., thigmotaxis), are more evident for the pre-escape compared to the pre-hunt condition. It also allowed us to investigate the movement trajectories involved in optimal hunting and escape.

We aimed to test several questions: (i) If the hypothalamus is involved in survival-related behavioral switches, we should be able to decode transitions between hunting and escaping using MVPA approaches; (ii) further we hypothesized that the hypothalamic connections with other regions, including the PAG, are involved in the switching between hunting and escaping; (iii) we next predicted that the hypothalamus is involved in the coordination of the behavior after the switching (e.g., coordinating escape behavior after the transition from the hunt). This would predict a stronger neural pattern indicating the switching would predict better movement coordination after the switching; (iv) finally, if the null hypothesis is correct, the hypothalamus is generally involved in switching between continuous movement behaviors, we expect to see the same in the control task.

Similar to the animal studies, we found that the human hypothalamus encodes the switching between hunting and escaping behavior in the MVPA analysis. Further, we show that the hypothalamus-PAG circuit and the hypothalamus-amygdala circuit are involved in the encoding of survival switching. However, human survival switching also involves network-scale interaction between regions known for “cognitive” task switching and survival behavior, including prefrontal cortical regions and the hippocampus. Model-based fMRI analyses showed that a strong hypothalamic multi-voxel pattern of switching, especially when it is synchronized with the multi-voxel pattern of switching of the amygdala is predictive of an optimal behavioral coordination, suggesting the involvement of hypothalamus–amygdala circuit in the motor coordination after the switching. Finally, in a control task, we did not observe ta role of the hypothalamus in switching between continuous behaviors, nor did it not interact with other regions to encode the switching, suggesting that the hypothalamus is likely to be involved in a switching between survival behaviors rather than being involved in a switching between general continuous behaviors.

## Results

In Expt. 1, we behaviorally tested 277 Prolific participants (details in S1 Text). In Expt. 2, we scanned 21 participants with fMRI as they performed 4 runs of a novel hunting-escape switching task inside the scanner. Participants were scanned for approximately 4 h each over 2 days (a total of 484 trials). Subjects controlled their avatar (player; yellow circle in Fig 1A) that was placed in a 2D circular field (Fig 1A). Subjects either hunted the computer agent (virtual prey; cyan circle in Fig 1A) or escaped from the computer agent (virtual predator). Subjects were shown a virtual arena with either a pink or orange boundary for 1 s, the color of which signaled the type of the next task (escape or hunt). The boundary color screen constitutes the Switch/Stay screen. One boundary color signaled the “Hunt Condition,” where the participants should chase and attempt to capture a virtual prey that was programmed to run away from them. The other boundary color signaled the “Escape Condition,” where the participants should prepare to escape a virtual predator which was programmed to attack them. Additionally, in Expt. 2 participants completed the control task to test whether hypothalamus involvement is specific to the survival behavior switching or is related to a general switching of the continuous behaviors (see Experimental paradigm for the control task in Methods section).

**Fig 2:**
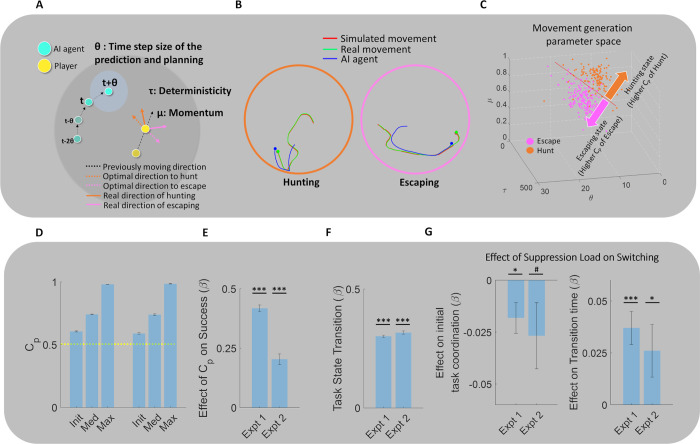
Computational model of behavior. (A) In the winning model, the subject first made a prediction about the future position of the computer agent (virtual predator or prey) based on the computer agent’s previous location, velocity, and acceleration. The subject then made a movement decision toward the direction of the predicted location of the prey during hunting behavior or made a movement decision away from the direction of the predator during escaping behavior. Unit time step *θ* controls how frequently the player makes computation to adjust their movement *τ* controls the deterministicity of the movement decision toward direction that minimizes the cost function, and the μ, a momentum parameter, controls the consistency of the movement between each movement decision. (B) Simulation results of player’s movement trajectory using a computational model. The trajectory of the simulated player’s movement was similar to the original player’s movement in both online (left; movement using the keyboard) and fMRI study (right; movement using the joystick). (C) Task-specific internal movement generation process. The current task state was decoded with high accuracy (>88% in both online and fMRI studies by using model parameters representing different components of movement generation processes, showing that the internal movement generation process is highly specific (red dashed line represents the SVM hyperplane which separates Escape (pink dots) and Hunt (orange dots) conditions). Condition-specific state (C_p_) was defined to represent a degree of the current condition-specific internal movement generation process. This was computed using the SVM decoder output, such that a farther distance from the hyperplane to the appropriate direction of the current task means higher C_p_. For example, in the hunting task, C_p_ becomes higher as the internal state goes farther in the direction of the orange arrow. (D) Change of the condition-specific movement generation state in one trial. C_p_ is low at the start of the experimental task. (E) Relationship between condition-specific internal generation state and success in each trial. In the logistic regression, higher C_p_ significantly predicted success in both online and fMRI studies. (F) Transition effect after switching. Condition-specific states significantly changed after switching. (G) Effect of suppression load on switching. High Condition-specific state (high C_p_ of the previous trial) interfered with initial task movement coordination after switching (left) and increased time to achieve stable movement coordination (right). ***p < 0.001, *p < 0.05, #*p* < 0.1. The data underlying this figure is available from https://osf.io/k5p3m/. SVM, support vector machine.

**Fig 3:**
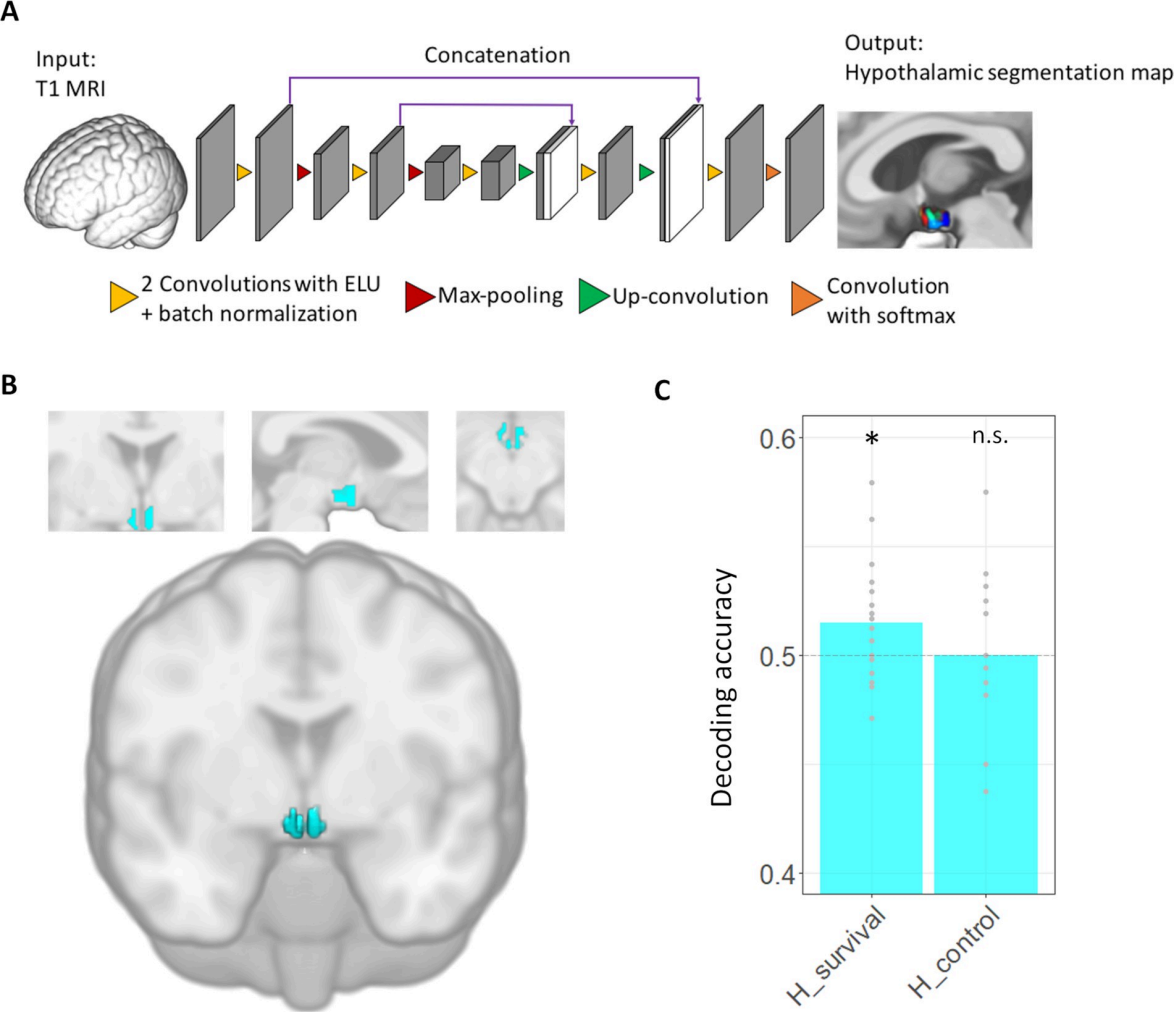
Hypothalamic segmentation and decoding accuracy. (A) CNN-based segmentation of the hypothalamus. Individual-specific segmentation of the hypothalamus and its subunits was done using the CNN-based segmentation algorithm of Billot and colleagues, which showed superior accuracy to atlas-based segmentation and manual segmentation. The input was an individual T1 image and the output was an image containing the segmentation of 5 subunits of the hypothalamus. **(B) Hypothalamic ROI in our study.** Among 5 subunits, hypothalamus ROI was defined by concatenating Posterior, Inferior tubular, and Superior tubular subunits which contain lateral and ventromedial hypothalamus that is known to encode hunting and escape [12,13]. **(C) Decoding accuracy of switch/stay in the hypothalamus**. The hypothalamus only encoded switching between survival tasks (escape vs. hunting) but not in switching between the control tasks. *p < 0.05. The data underlying this figure is available from https://osf.io/k5p3m/. CNN, convolutional neural network; ROI, region of interest.

We first examined the characteristics of escaping and hunting behavior in terms of the difference in participants’ movement patterns and preparation behavior. Then, we fit a computational model that explained the generation process of both escaping and hunting behavior (Fig 2A) and tested the computational mechanism of transitions in behavioral generation after switching by using this model. At the neural level, we first asked whether the hypothalamus encodes Switch/Stay information and how hypothalamic interaction with other regions is involved in Switch/Stay information. We performed MVPA on nine ROIs including the hypothalamus (see ROI selection and segmentation for MVPA analyses in Methods section; Fig 3A and 3B) and then performed Informational Connectivity analysis [23] between the 9 ROIs to show how the hypothalamus interacts with other regions to encode switching of survival behaviors. MVPA analyses were also conducted for the control task to compare survival switching with general attentional-motor switching. Finally, by using a generative model of behavior, we investigated whether and how the hypothalamus initiates the coordination of the survival behavior after switching in conjunction with other regions. Experiments 1 and 2 were preregistered (https://osf.io/ge8c3 and https://osf.io/kx5af).

### Movement characteristics of hunting and escaping

The average success rate over all the conditions (Escape and Hunt) was 54.8% in Expt. 2 (53.6% in Expt. 1) and the success rate was higher in the Escape condition than in the Hunt condition (59.0% versus 50.7% in Expt. 2, 56.1% versus 51.0% in Expt. 1; *p* < 0.001 in paired *t* test of success rate difference between the 2 conditions; preregistered), despite that we applied the same algorithms to allow subjects to achieve a success rate of approximately 50%. Pre-encounter behavioral analysis showed that participants made more thigmotaxic movements in the pre-escape condition compared to the pre-hunt condition (0.52 versus 0.23, t[276] = 27.06, *p* < 0.001 in Expt. 1; 0.41 versus 0.18, t[20] = 5.85, *p* < 0.001 in Expt. 2; preregistered), showing that participants exhibited more anxiety-like behaviors and understood the experimental context (meaning of the boundary colors). There was a significant difference in the average degree of direction change per unit of time, with more variability in the Escape condition (paired *t* test for movement variability difference between escaping versus hunting; 19° versus 9°, t[276] = 23.77, *p* < 0.001 in Expt. 1; 18° versus 9°, t[20] = 7.44, *p* < 0.001 in Expt. 2; exploratory). Movement direction followed a less predictable direction in the Escape condition compared to the Hunt condition (linearity: 0.55 versus 0.35, t[276] = 21.90, *p* < 0.001 in Expt. 1; 0.58 versus 0.45, t[20] = 13.19, *p* < 0.001 in Expt. 2; preregistered), which is consistent with previous studies showing that movement direction of evasion behavior is less predictable than pursuit behaviors [26]. We tested whether behavioral differences between hunting and escaping were affected by “boosting” moments by comparing movement linearity and variability between escape and hunt conditions, excluding time points when the predator “boosted.” Results were consistent after excluding those time points: the hunt condition showed higher movement linearity (t[276] = 21.89, *p* < 0.001 in Expt. 1; t[20] = 10.24, *p* < 0.001 in Expt. 2), while the escape condition had higher movement variability (t[276] = 23.27, *p* < 0.001 in Expt. 1; t[20] = 6.88, *p* < 0.001 in Expt. 2). However, there is a possibility that the introduction of boosting exaggerated or decreased the behavioral difference between the 2 conditions. Success rates in the control task were 58.2% (averaged over all conditions), 60.1% (descending order task), and 56.3% (ascending order task).

We also tested the change in success rates after the Switch compared to the Stay condition. The success rate was decreased in the Switch condition compared to the Stay condition both in the experimental task and the control task (success rate after Switch versus Stay: 0.55 versus 0.52, t(276) = 6.95, *p* < 0.001 in the experimental task of the Expt. 1; 0.57 versus 0.53 t[20] = 2.86, *p* = 0.0097 in the experimental task of the Expt 2; 0.65 versus 0.53, t[14] = 6.53, *p* < 0.001 in the control task).

### A generative model of hunting and escaping behavior and condition-specific internal movement generation process

We tested 3 computational models of the hunting and escaping behavior (M1, M2, and M3; preregistered) where in M2 and M3, an agent utilizes their internal predictive model of virtual prey/predator’s movement to guide their escaping of hunting behavior while in the M1, an agent only utilizes current location information of the virtual prey/predator to make movement decision. In the winning model (M3 in Fig 2A) according to the Bayesian model comparison (Protected Exceedance Probability = 1 in both Expts. 1 and 2; mean BIC for M1, M2, M3: 58.8, 59.7, 21.3 in Expt. 1, and 140.8, 140.9, 73.7 in Expt 2; S1A Fig), subjects first made a prediction about the future position of the computer agent (virtual predator or prey) based on the computer agents’ previous location, velocity, and acceleration information (see below equation and Methods section for details). Then, subjects made a movement decision toward the direction of the predicted location of the prey (*y*_*pred*_ in the below equation) during hunting behavior or made a movement decision away from the direction of the predator during an escaping behavior (Fig 2A; simulation results for this model, Fig 2B).


$$y_{pred}(t)=y(t)+y\prime (t)*\theta +y\prime \prime (t)*\frac{1}{2}\theta^{2}$$

Note that hunting and escaping behavior was controlled by the same sets of parameters (θ, τ, μ). The unit time step parameter θ (possible ranges between 0 and 30; 30 is the length of the model fitting time window; S1 Table and S1B Fig) controls the degree of the prediction (higher θ will cause more predictive movement as an agent will predict more distant future position) as well as the frequency of movement decision. In an M2 model, θ was fixed to 1 meaning that the degree of prediction and computation is invariant though an agent utilizes prediction. The τ (possible ranges between −1,000 and 1,000; S1 Table and S1B Fig) controls the deterministicity of the movement decision toward the optimal direction that minimizes the cost function (high τ: high probability of movement decision to the optimal direction), and the μ (possible ranges between 0 and 1; S1 Table and S1B Fig), a momentum parameter, controls the consistency of the movement between each movement decision (whether subjects consistently move toward the direction decided in time step k step before making new computation in the next time step k+1). This enables us to directly compare the movement generation process between hunting and escaping behavior.

Similar to the behavioral analyses, in both Expts. 1 and 2, we found that internal movement generation process of escaping and hunting is different in terms of model parameter settings (Materials and methods) and we were able to decode whether the current task state is hunting or escaping from model parameters with high accuracy (88.7% in Expt. 1; 90.0% in Expt. 2; all *p* < 0.001 in *t* test against chance level; all decoding analyses of parameters are exploratory) which means that movement generation states are highly specific to each survival behavior. Then, we quantified the Condition-specific state (C_p_), representing how the current movement generation state is specific to the current survival behavior using the decoder output (e.g., if the decoder output is 80% hunt/20% escape and the current trial is Hunt condition, C_p_ = 0.8). C_p_ = 1 means that the current state is specialized for the current task (e.g., hunting in the Hunt condition) while C_p_ = 0 means that the state is specialized for the inappropriate task (e.g., escaping in the Hunt condition). C_p_ = 0.5 means the state is undifferentiated for neither of the tasks. We expected that this condition-specific internal movement generation process would enable appropriate task behavior, which was reflected in increased success rate by the average C_p_ of the trial (beta = 0.42 and 0.2 in Expts 1 and 2, respectively, all *p* < 0.001; Fig 2E).

We then explored the characteristics of the condition-specific internal movement generation process. C_p_ at the start of each trial was significantly condition-specific, but was still close to the undifferentiated state (0.61 and 0.59 in Expts. 1 and 2; all *p* < 0.001 in *t* tests against the undifferentiated state; Fig 2D) and increased to achieve the Condition-specific state in the later times (median C_p_ of 1 trial = 0.74 and 0.74 in experiments 1 and 2; Fig 2D). Also, we observed the change of condition-specific movement generation state after switch by testing the change of C_p_ toward the direction of the current task from the previous task (e.g., from escaping-specific state to hunting-specific state; change of C_p_ toward current task = 0.3 and 0.32 in experiments 1 and 2, respectively, all *p* < 0.001 in one-sample *t* test, Fig 2F).

Previous studies showed that the task-switching process involves both the initiation of the new task and/or the suppression of ongoing task representation [27,28]. Therefore, we next asked (i) whether the switching process involves both suppression of the previous movement generation process (e.g., escaping) and the coordination of the new task; or (ii) whether the switching process only involves the coordination of the new task. If the former is true (suppression and coordination), switching to the new task would be more difficult if the previous task requires stronger suppression. Therefore, we defined the suppression load of the switching as the C_p_ of the previous trial before switching, since high C_p_ before switching means that the internal movement generation process was more biased into the previous task, which makes it harder to suppress.

We found that the high C_p_ of the previous trial (just before the switch cue), which would increase the suppression load, negatively affects the initial C_p_ of the current trial (beta = −0.02 and −0.03; *p* = 0.015 in Expt. 1; marginally significant with *p* = 0.092 in Expt. 2, respectively, Fig 2G). Furthermore, the high C_p_ of the previous trial also increased the time to achieve a Condition-specific state (beta = 0.03 and 0.02; *p* < 0.001 and *p* = 0.043 in Expts. 1 and 2, respectively, Fig 2G). These results support that the switching process not only involves the coordination of new tasks but also involves the suppression of the previous condition-specific movement generation process.

In summary, by using the computational modeling of the escaping and hunting behavior, we found that an internal movement generation process for each behavior is highly specific and these Condition-specific states are beneficial for successful escaping and hunting behavior. Furthermore, we found that the switching process involves both the suppression of the previous Condition-specific state and the coordination of the current Condition-specific state.

### The hypothalamus encodes switch and stay information of survival behaviors

**Fig 4:**
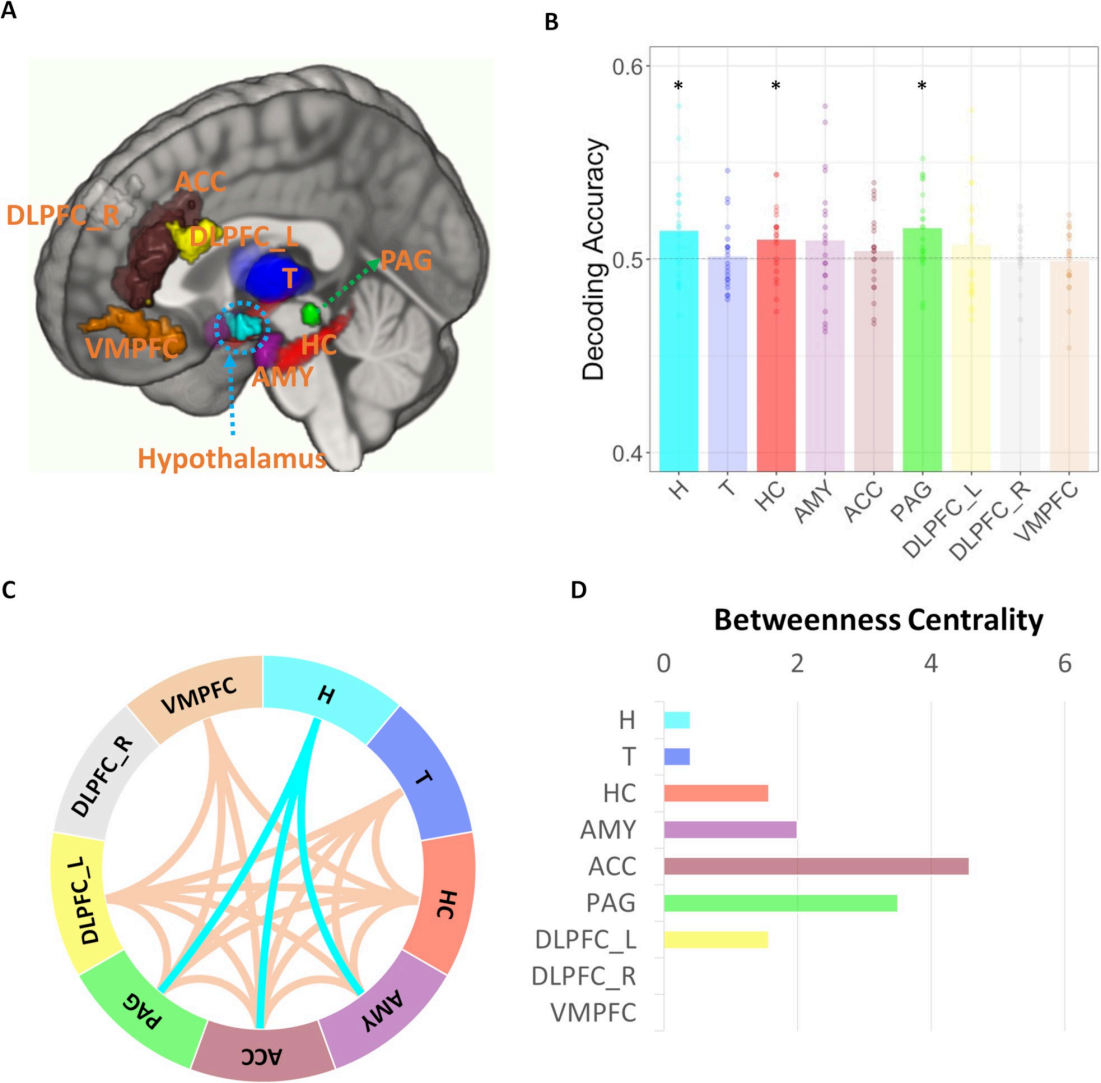
Region- and network-level encoding of Switch/Stay information. (A) ROIs used in this study. Those ROIs included the Hypothalamus (H), Thalamus (T), Hippocampus (HC), Amygdala (AMY), ACC, PAG, left and right dorsolateral prefrontal cortices (DLPFC_L and DLPFC_R), and vmPFC. (B) Decoding accuracy of switch/stay in ROI-based MVPA analyses. The hypothalamus, hippocampus, and PAG significantly encoded switch/stay information (pFDR < 0.05). (C) Multi-voxel functional connectivity (informational connectivity) between a priori selected ROIs. Informational connectivity was computed by using covariation trial-by-trial decoding accuracy between a pair of regions. This resulted in a connectivity matrix between ROIs. NBS revealed a dense network of regions encoding switch information. DLPFC_R was not included in this network (survival-related behavioral switching network). The hypothalamus was connected to ACC, PAG, and amygdala. (D) We computed BC within this network to find hubs connecting regions within the survival behavior switching network. The top three hubs based on BC were ACC, PAG, and amygdala which were connected to the hypothalamus. *p < 0.05. The data underlying this figure is available from https://osf.io/k5p3m/. ACC, anterior cingulate cortex; BC, betweenness centrality; MVPA, multi-voxel pattern analyses; NBS, network-based statistics; PAG, periaqueductal gray; ROI, region of interest; VMPFC, ventromedial prefrontal cortex.

Our main question was whether the hypothalamus encodes information regarding switching between hunting and escaping behaviors. MVPA analyses were performed on 9 ROIs (preregistered; except amygdala) that were associated with task switching or survival behavior in previous literature [9,13,27,29–31] (Fig 4A) using the COSMOMVPA toolbox pipeline [32]. We found that Switch/Stay information is significantly decodable in the hypothalamus (decoding accuracy: 51.5%; t(20) = 2.49, pFDR = 0.036, one-tailed, in a *t* test against chance level (50%), *p*-values were FDR corrected for 9 ROIs; Fig 4B). In addition to the hypothalamus, we found that PAG and hippocampus (HC) also encoded Switch/Stay information (decoding accuracy: 51.6% and 51.0% for PAG and HC, respectively; all pFDR < 0.05, one-tailed; Fig 4B). We also asked whether this encoding of switching information in the hypothalamus was specific to switching between survival behaviors (hunting versus escaping) or whether it generalized to other types of motor switching. Using the same MVPA analyses with the control task data, we found that the hypothalamus did not encode switch information in the control task (decoding accuracy: 50.0%; t(14) = −0.05, pFDR = 0.518, one-tailed; Fig 3C). The searchlight analysis of the control task showed a cluster in the DLPFC (S2A Fig; also see S2B Fig for the searchlight results of the experimental task). Note that we tested the possibility that this difference between the experimental task and the control task could be due to a difference in the sample size. We re-conducted the MVPA on the experimental task only using 15 participants’ data who completed the control task which showed similar results (S2 Text).

### Hypothalamic synchronization with PAG, amygdala, and ACC encodes switching between survival behaviors

The coordination of survival behavior requires interaction between multiple regions. For example, animal studies showed that lateral hypothalamic projection to PAG underlies the switching between predation and evasion [13,14] as well as the role of interaction between hypothalamus and amygdala in predation [33] and evasion [11]. Similarly, we expected that the switching of survival behaviors in humans should be associated with a network-level interaction including a hypothalamus-PAG interaction and a hypothalamus-amygdala interaction. To identify such networks, we measured inter-regional multi-voxel pattern synchronization associated with switching between escaping and hunting using Informational Connectivity [23,25,34,35] (Exploratory analyses). Like MVPA, an advantage of IC over univariate functional connectivity is that IC utilizes all patterns of responses within regions to encode information that is lost by averaging, which identifies functional connections that cannot be found in univariate functional connectivity analyses [23,24], including connections with small brainstem nuclei [36]. Furthermore, IC allows us to test regional interactions in terms of specific experimental conditions in [24] such as switching between escaping and hunting in our study.

IC was measured between every pair of the 9 ROIs and the network-based statistics (NBS) revealed a network whose multi-voxel pattern synchronization changes with switching between escaping and hunting (survival behavior switching network; Fig 4C and 4D). In other words, information content (voxel patterns) in regions of the survival behavior switching network were connected. A total of 8 regions except right dorsolateral PFC (DLPFC_R) were included in the survival behavior switching network and they were densely connected, showing a network-level encoding of the switching between survival behaviors. Note that although the amygdala, thalamus, vmPFC, and ACC did not encode Switch/Stay in an MVPA analysis, information in these regions (multi-voxel pattern) was connected to ROIs encoding Switch/Stay at a regional level.

Then, to find hubs connecting regions within the survival behavior switching network, we computed the betweenness centralities of each region of the survival behavior switching network which represent the fraction of all shortest paths that contain a specific node. The top 3 hubs based on BC were ACC, PAG, and amygdala, and the BC of VMPFC was 0 which means this region does not contain any shortest path that connects other regions. Interestingly, the hypothalamus was connected with the top 3 hubs (Fig 4C and 4D), which is consistent with previous animal studies showing hypothalamic interaction with the PAG and amygdala. These results also suggest a possibility that the hypothalamus might have communication with regions of the survival behavior switching network through these hubs. Additionally, in a whole brain seed-based IC analysis using hypothalamus as a seed, we found one very small cluster in the right middle temporal lobe (pTFCE < 0.05).

IC analyses in the control task revealed a network that encodes motor switching in the control task composed of the thalamus, hippocampus, amygdala, ACC, and the bilateral DLPFC, which network did not include the hypothalamus (see S2 Text for details; S3 Fig). Interestingly, similar to the survival behavior switching network, ACC was the hub region of switching in the control task, suggesting the ACC’s role in the general switching process.

### The hypothalamic switching signal predicts the coordination of optimal behavior after switching

We showed that the hypothalamus encodes Switch/Stay information in MVPA analyses. Then, what is the role of the hypothalamus in the switching process? Animal studies show the role of the hypothalamus and PAG in switching between predation and escape [13], as well as the coordination of those behaviors after survival switching [14]. Thus, we expected that the hypothalamus and PAG would be involved in the coordination of survival behavior after switching in humans. We expected that the strength of neural signaling indicating switching of survival behaviors measured by a multi-voxel pattern strength of switching (MVPSS; defined using a decoder output strength in MVPA analyses with a range between 0 and 1; 1: 100% switch; 0: 0% switch, same as 100% stay) in the hypothalamus and PAG would facilitate the condition-specific internal movement generation of the following task after switching (Exploratory analyses).

**Fig 5:**
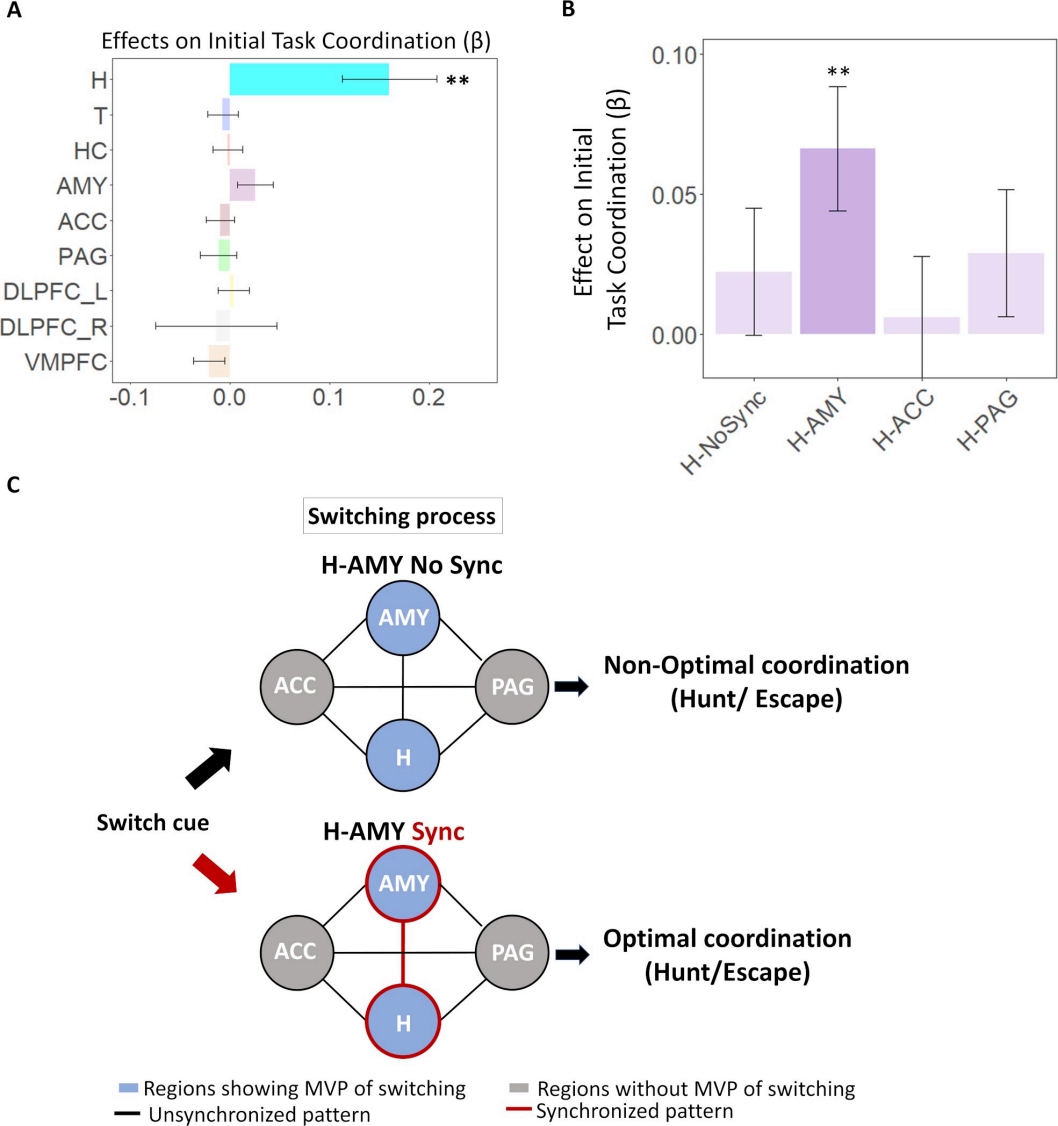
Movement optimization based on Hypothalamic switching pattern and pattern synchronization with other regions. (A) The regional multi-voxel pattern of switching (MVPSS) predicts condition-specific movement coordination after switching. In the regression analyses, only hypothalamic MVPSS significantly predicted optimal condition-specific movement coordination after switching (pFDR < 0.01). (B) Regions connected to the hypothalamus. (C, D) Switching pattern synchronization between the hypothalamus and connected regions predicts optimal behavior coordination. The hypothalamic multi-voxel pattern of switching (hypothalamic MVPSS) predicted optimal task coordination only when there is a simultaneous multi-voxel pattern of switching in the amygdala (*p* = 0.003; H-AMY Sync in Fig 5D). Hypothalamic MVPSS was irrelevant with optimal behavior coordination when there was synchronization with connected regions other than the amygdala (H-ACC Sync or H-PAG Sync in Fig 5D) or there was no synchronization with any of the connected regions (H-NoSync in Fig 5D). See Fig 5C for the schematic explanation. **p < 0.01. The data underlying this figure is available from https://osf.io/k5p3m/. ACC, anterior cingulate cortex; MVPSS, multi-voxel pattern strength of switching; PAG, periaqueductal gray; VMPFC, ventromedial prefrontal cortex.

We found that among the nine ROIs, only the hypothalamic MVPSS predicts the initial C_p_ of the trial after switching (mixed-effect regression to predict initial C_p_ after switch by using MVPSS of the hypothalamus; beta = 0.18, t = 3.76, *p* = 0.0002; Fig 5A), while all other 8 ROIs including the PAG and hippocampus that encoded Switch/Stay in MVPA analyses were not associated with task behavior coordination (all *p* > 0.12; Fig 5A), meaning that only the hypothalamic switching signal, but not the switching signals from other regions, is associated with the coordination of the condition-specific behavior after switching. However, hypothalamic MVPSS did not predict initial C_p_ after stay behavior (*p* = 0.6243), showing that the MVPSS is not associated with a continuation of coordination of ongoing task behavior, but is instead associated with coordination of the new task behavior after switching. Furthermore, a strong hypothalamic MVPSS also predicted success in each trial (beta = 0.11, t = 2.06, *p* = 0.039; a mixed-effect logistic regression to predict trial-by-trial success in the experimental task) while it did not predict success in the control task (beta = −0.06, t = −1.24, *p* = 0.215), supporting that the hypothalamus is more actively involved in survival behavior switching along with the MVPA results. Lastly, the hypothalamic MVPSS predicted a shortening of the time to achieve stable task coordination (time to achieve C_p_ = 60 after switching; beta = −0.12, t = −3.23, *p* = 0.001), which also supports that the hypothalamic switching signal is associated with the facilitation of the condition-specific behavior coordination after switching.

We next tested whether the hypothalamic influence on coordination of the initial behavior after switching is associated with the suppression of the previous behavioral coordination or is associated with initiating a new condition-specific behavior. First, we showed that the degree of condition-specific behavioral coordination in the previous trial (last C_p_ of the previous trial) does not predict the MVPSS of the hypothalamus, supporting the claim that the hypothalamic switching signal is not associated with the suppression of the previous task coordination (*p* = 0.8864). Second, in an additional regression model to predict initial C_p_ by using both MVPSS of the hypothalamus and the last C_p_ of the previous trial, we found that MVPSS of the hypothalamus significantly predicted the initial C_p_ (beta = 0.17, t = 3.38, *p* = 0.0007) after regressing out the effect of the last C_p_ of the previous trial, which affected initial C_p_ negatively with marginal significance (beta = −0.03, t = −1.85, *p* = 0.064). These results suggest that the hypothalamic switching signal affects initial condition-specific behavior coordination after switching that is independent of suppressing previous task coordination.

### Hypothalamus-amygdala synchronization is required for behavioral coordination

Previous studies consistently showed that coordination of survival behavior requires interactions between multiple regions such as an interaction between the hypothalamus, PAG [13,14], and amygdala [37] and we showed that these regions (hypothalamus, PAG, and amygdala) show synchronization related to switching of survival behaviors. Then, the next question is, if the hypothalamic switching signal is involved in the coordination of optimal behavior after switching, does this coordination depend solely on the hypothalamus or does it require interaction between the hypothalamus and connected regions?

To answer this question, we classified the hypothalamic switching signal into 4 cases. The first case is a hypothalamic switching signal without synchronization with other regions (1 if hypothalamic multi-voxel pattern indicates switching and other connected regions’ multi-voxel patterns (that is, PAG, ACC, and amygdala) are non-switching pattern; otherwise 0), and the other 3 cases are hypothalamic switching signal synchronized with switching signal of connected regions (e.g., for the PAG case, 1 if both hypothalamic and PAG multi-voxel pattern indicates switching while patterns of ACC, and amygdala is non-switching; 0 otherwise). We ran 4 regressions to test which case is associated with optimal behavioral coordination after switching (exploratory analysis). Results showed that optimal coordination was enabled only when there is a hypothalamic switching signal synchronized with the switching signal of the amygdala (beta = 0.06, t = 3.18, *p* = 0.0015; Fig 5B and 5C) while the hypothalamic switching signal without synchronization nor synchronization with ACC/PAG was not associated with optimal coordination (*p* > 0.15; Fig 5B).

Additionally, given that different amygdala subnuclei play different roles, we tested which nuclei play a more active role in conjunction with the hypothalamus (see the S3 Text for details; exploratory analysis) and we found that the hypothalamus-centromedial amygdala (CMA) synchronization (beta = 0.02, t = 1.99, *p* = 0.047), but not the hypothalamus-basolateral amygdala (BLA) synchronization (*p* = 0.341) predicts optimal movement coordination after the switching. We also tested whether each of the amygdala subnuclei encodes switching itself or if they directly predict optimal movement coordination after the switching without synchronization with the hypothalamus (please see the S3 Text for details).

## Discussion

The role of the hypothalamus in survival behavior has been extensively investigated in non-human animals [11–14] while the evidence supporting the role of the human hypothalamus in survival behavior is very rare. In this preregistered study, we showed the human hypothalamus coordinates switching between survival behaviors by introducing a novel ecologically valid experimental paradigm and by optimizing the acquisition and analyses of hypothalamic BOLD signal. MVPA results showed that the hypothalamus encodes switching between hunting and escaping behaviors as in other animal studies and this hypothalamus is connected to PAG, amygdala, and ACC within the survival behavior switching network. Importantly, by adapting computational modeling of continuous escaping and hunting behavior, we found that the hypothalamus is the only region in the survival behavior switching network that is associated with facilitating the coordination of survival behavior after switching and this function of the hypothalamus requires synchronization with the amygdala.

Behaviorally, we utilized an ecologically valid experimental paradigm that resembles tasks in animal studies of survival behavior [13,16]. In both Expts. 1 and 2, we showed both similar and divergent computational mechanisms underlying human hunting and escaping behavior. The winning model showed that participants made predictive movements by a computation of future prey/predator position both in hunting and escaping behavior, which extends previous studies showing predictive predation behavior in the macaque [16]. On the other hand, model parameter comparison also showed that the behavioral generation process of hunting and escape is highly divergent and specific to each state (decoding accuracy >88%). Compared to hunting behavior, escaping behavior was more stochastic which is consistent with other modeling results in other species [26]. The unit time step of movement decision in escaping behavior was also shorter. These stochastic and short-time step decisions make escaping movements more unpredictable to the predator and enable more flexible coordination of behavior to avoid the predator (e.g., by frequent directional change). We also explored the computational mechanisms of switching between escaping and hunting by defining the degree of the Condition-specific state (C_p_). Higher C_p_ was associated with successful hunt/escape and switching also induced appropriate change of Condition-specific state. Furthermore, we showed that successful transition is affected by the suppression load of the previous trial, showing that similar to the classical task switching process, survival switching also requires suppression of the ongoing task process [27,28].

To our knowledge, this is the first human study showing the role of the hypothalamus in switching and coordination of survival behavior. Small size, closeness to ventricles, and low contrast to adjacent tissues were the obstacles to the precise identification of the hypothalamus and its subnuclei. We segmented the hypothalamus at the level of subnuclei by applying state-of-the-art deep learning-based segmentation [19] that outperforms conventional manual segmentation [38] and atlas-based segmentation [39]. The hypothalamus is composed of numerous different nuclei with largely heterogeneous functions such as thermoregulation in the anterior hypothalamus [40], as well as circadian rhythm regulation in the suprachiasmatic nucleus [41]. Thus, the inclusion of hypothalamic subregions that are not related to survival behavior would decrease the sensitivity of detection in our study. Therefore, by applying subnuclei-level segmentation of the hypothalamus, we constructed a hypothalamic ROI focused on the lateral and ventromedial hypothalamus that were previously identified as key hubs of survival behavior in animal studies [12,13]. We excluded other parts of the hypothalamus that have been less relevant to animal survival behavior. In addition, we regressed out CSF and other potential confounders and used a short TR for better statistical modeling of the hypothalamic signal. MVPA after these processing steps showed that the human hypothalamus indeed encodes switching between hunting and escape, as in animal studies [13,14]. Unlike in the survival behavior switching in the experimental task, the hypothalamus was not involved in the control task. Instead, using searchlight MVPA analyses, we found a small cluster in the DLPFC that encodes motor switching, which is consistent with previous studies of non-survival task switching [27,42]. Furthermore, the IC analysis of the control task showed that network encoding the switching in the control task does not contain the hypothalamus (S3 Fig and S2 Text). Lastly, the hypothalamic MVPSS in the control task did not predict success in the trial after the switching. These results support that the hypothalamus is more likely to be involved in the switching of survival behaviors rather than involved in the switching of general continuous behaviors. Lastly, it could be possible that the hypothalamic involvement in switching found in our study might be related to a change of arousal or emotional valence (see S4 Text for the detailed discussion). However, since we concatenated both switching from hunt to escape and switching from escape to hunt it is less likely that the difference in emotional valence or arousal level between hunt and escape influenced the hypothalamic switching signal. Also, we showed that the electric shock that increases arousal was not associated with the hypothalamic switching signal (S4 Text).

We also found that the PAG encodes the switching with the hypothalamus and hypothalamus-PAG multi-voxel activation patterns of switching are functionally coupled. Previous studies consistently showed the importance of hypothalamus-PAG coupling in survival behavior [6,13,14,43]. For example, Li and colleagues showed that an activation of PAG-projecting GABA neurons in the lateral hypothalamus drives predatory attack in mice while an activation of PAG-projecting glutaminergic neurons drives evasion [13]. Recently, Rossier and colleagues further showed that PAG-projecting GABA neurons of the lateral hypothalamus block defensive response encoded in the PAG in mice [14]. The ventromedial hypothalamus also heavily projects to the PAG and coordinates survival behavior [44]. However, unlike previous animal studies, neither the strength of the switching signal in the PAG quantified by MVPSS nor hypothalamus-PAG pattern synchronization were directly associated with optimal movement coordination after switching in our study. Hypothalamic MVPSS and hypothalamus-amygdala pattern synchronization were associated with optimal movement coordination. Unlike previous animal studies, our results showed that the neuronal pattern of switching between hunting and escaping in humans cannot be entirely explained as an interaction between the hypothalamus and PAG, which known as a “reactive” survival circuit that mainly utilizes the PAG as a motor pattern generator for pre-programmed behavior but also involves an interaction between classic the hypothalamus-PAG circuit and the regions involved in “cognitive switching” area that previously known to encode the switching of abstract task representation [27]. These regions include DLPFC, thalamus, hippocampus, and ACC [27,45]. This claim is supported by our computational modeling results suggesting that subjects utilized an internal model of the predator/prey to make predictive escaping/hunting movements. Also, note that experimental context in previous animal experiments usually includes an interaction with close predator/prey which requires a reactive behavior [10,13,44]. Previous animal studies show that the hypothalamus-PAG circuit is required for both simple/reactive and complex/cognitive survival behavior [11]. However, in a “reactive” context, the PAG acts as a direct motor pattern generator such that the direct stimulation of the PAG can produce pre-programmed survival behavior [11]. On the other hand, in a “cognitive” context, the hypothalamus-PAG circuit was necessary but not sufficient such that the direct stimulation of the PAG could not generate the strategic survival behavior under a sophisticated survival situation [11], which is consistent with our results showing significant involvement of the hypothalamus-PAG circuit in encoding switching without direct association between PAG and movement coordination. To our knowledge, the role of PAG in this “cognitive” context is still ambiguous, but one recent study suggested its role in increasing cognitive control [46].

In addition to the hypothalamus and PAG, MVPA results showed that the hippocampus encodes switching, which is a critical region of model-based planning [6] and the flexible encoding of the novel context by suppressing the old context [47]. Furthermore, we found a survival behavior switching network which was a network having dense interaction between multiple regions. Unlike in previous animal studies of reactive task switching, this survival behavior switching network not only involved hypothalamic-PAG interactions but also included interaction between (i) regions known for “cognitive” task switching such as DLPFC (left) [42], thalamus [27,48], and ACC [49]; and (ii) cognitive fear circuit regions including hippocampus and ventromedial prefrontal cortex [9] that is associated with strategic and deliberate survival behavior; and (iii) amygdala. The thalamus is known to control task representation in the PFC such that the mediodorsal thalamus suppresses task-irrelevant representation while augmenting task-relevant representation [27,48]. The ACC helps increase cognitive control when there is an increased need for control, as in a situation involving conflict [50] or task switching [49], by computing control demands from the integration of information from multiple regions [50]. In our study, ACC was the hub that connected all regions within the network both in the main task and in the control task. Overall, these findings again support our claim that switching between human survival-related behavior requires recruitment of a “cognitive” task survival behavior switching network and behavioral coordination network in addition to the hypothalamus-PAG circuit that is necessary for survival behavior regardless of the complexity of behavior.

A lingering question is what is the specific role of the hypothalamus in survival switching? Among the regions of the survival behavior switching network, the hypothalamus was the only region that was directly associated with movement coordination after switching such that a strong hypothalamic switching pattern was associated with higher condition-specific movement coordination after switching. However, hypothalamic MVPSS was not affected by the suppression load of the previous trial. This suggests that the hypothalamus might be a region that initiates motor pattern generation after switching while other regions are more involved in non-motor and abstract parts of the switching process, such as integrating sensory information, encoding emotional states, suppressing old task representations, and building a new task representation. Although evidence in human studies supporting the hypothalamus as a survival motor coordination is very rare, many animal studies point to the hypothalamus as a movement initialization region [10,13,14]. The hypothalamus was connected to the PAG, amygdala, and ACC and we found that the hypothalamic switching signal is associated with motor coordination only when there is a switching signal in the amygdala. Especially, among the amygdala subnuclei, only the synchronization with the CMA, but not the synchronization with the BLA was associated with the motor coordination.

Currently, the known role of the amygdala in defensive behavior, especially the central amygdala, is that it integrates sensory cues to represent the current internal state and broadcasts that state to other brain regions including motor pathways, which can enable, state-dependent behavioral responses [31,51]. Furthermore, the amygdala is the upstream of the hypothalamus [52] and the projections from the central amygdala to the hypothalamus coordinate both aggression and avoidance [53,54] as well as controlling approach and avoidance [55]. Based on these prior studies, and our findings showing hypothalamic switching signal synchronized with the amygdala, especially CMA, to predict optimal motor coordination while the switching signal of the amygdala itself did not affect motor coordination, we speculate that the amygdala processes the sensory cue information that informs the switching or staying of ongoing behavioral states and the hypothalamus receives this information to produce appropriate task behavior after switching. However, we note that we cannot determine a causal relationship between the hypothalamus and amygdala in this study and there is also a possibility that the hypothalamus is an input region to the amygdala that coordinates complex survival behavior after switching.

In contrast to the hypothalamus, the hippocampal MVPSS was associated with the suppression load of the previous trial but not with the current C_p_, suggesting its potential role in suppression rather than motor pattern generation in survival-related behavioral switching. This is consistent with previous literature showing the hippocampus’ role in inhibiting the old context, especially through an interaction with the prefrontal cortex [47,56], which aligns with the hippocampal interactions with DLPFC_L and VMPFC during switching in our IC analyses. However, the role of these interactions in survival-related behavioral switching remains unclear in our study, and this would be an interesting topic for future studies. Although our results provide some indirect evidence of the potentially different roles of the hypothalamus and hippocampus in survival-related behavioral switching, our current task does not clearly distinguish between the suppression-related process and motor coordination independent of the suppression-related process. Therefore, future studies with tasks that clearly differentiate between suppression and coordination within the switching process (for both the survival-related main experimental task and the control motor task) will be needed to further address this question.

Lastly, in this study, we demonstrated that the human hypothalamus plays a role in switching between hunting and escaping, interacting with cognitive task-switching regions. While the hypothalamus was not implicated in the control task, suggesting it may not be part of the classic “domain-general” switching network involving the DLPFC and ACC, which is known to be involved in various domain switches such as reversal learning and set-shifting [45], our findings, when combined with existing animal literature, suggest that the hypothalamus may be involved in switching between general human survival-related states. These states encompass brain functions closely tied to maintaining homeostasis and reproduction [7]. For example, animal studies have shown that the hypothalamus controls switching between mounting and attacking, as well as between feeding and seeking social contact [12,57]. While human survival-related behavior occurs in more complex social contexts requiring intricate, strategic planning compared to innate animal behaviors, we propose that the hypothalamus may interact with both cognitive task-switching areas and brain networks coordinating complex social interactions to facilitate switching between complex survival states. This area presents an intriguing avenue for future human experimental studies.

In conclusion, we found that the human hypothalamus encodes switching between hunting and escaping behavior. Similar to animal studies, the hypothalamus-PAG circuit was involved in the encoding of switching. However, human survival switching also involved network-scale interaction between regions that have been known for “cognitive” task switching and survival behavior, and this was consistent with our behavioral findings showing our human subjects utilized internal predictive models of prey and predator to coordinate their hunting and escaping movement, respectively. Finally, the hypothalamus was the only region within the survival behavior switching network that was associated with optimal survival coordination which required a synchronization with the amygdala which we posit plays a role in integrating sensory information to represent the current state and conveys this information to the hypothalamus. These findings extend our understanding of the human hypothalamus from a region that regulates our internal bodily states to a region that switches survival behaviors and coordinates strategic survival behaviors.

## Materials and methods

### Participants

In experiment 1 (Caltech IRB No.: 20–0978), we used the online experimental platform Prolific to examine behavior and build our computational model. We included 277 participants (male: 179, female: 92, gender unspecified: 6; age: 18 to 65, median age: 24.0) according to the following criteria: (i) age between 16 and 65; (ii) self-reported normal or corrected-to normal-vision; (iii) fluent English speaker; (iv) previous approval rate above 90%; (v) completed more than 80% of total trials; and (vi) correct computer and monitor setting (strong internet connection with 60 Hz monitor). In experiment 2 (Caltech IRB No.: 16–0683), 22 participants performed 4 runs of the hunt-escape switching task inside the MRI scanner. Participants were scanned for approximately 4 h each over the course of 2 days (2 runs for each day). One participant’s data was excluded since we failed to acquire an MR signal from the hypothalamus, resulting in the reporting of 21 participants for behavioral and neural data analyses. All participants provided written informed consent to participate in the study, which was reviewed and approved by the Committee for the Protection of Human Subjects (IRB) at the California Institute of Technology. Both experiments were conducted according to the principles expressed in the Declaration of Helsinki.

### Experimental paradigm

#### Experimental paradigm for the experiment 2

Subjects controlled their character (subject; yellow circle in Fig 1A) that was placed in a 2D circular field (Fig 1A). Split across 484 trials (121 trials per each run; 4 runs of task), subjects asked to either hunt the computer agent (virtual prey; cyan circle in Fig 1A) or escape from the computer agent (virtual predator).

Step 1: Subjects are shown a virtual arena with either a pink or orange boundary for 1 s, the color of which signals the type of the next task. This constitutes the Switch/Stay screen (480 Switch/Stay screen across 4 runs; 120 switch/stay screens per run). This explicit Switch or Stay screen between trials was used as a time window to decode the Switch versus Stay using the MVPA with minimal movement-related BOLD signal since participants did not move the Switch or Stay screen. The orange boundary signals the “Hunt Condition,” where the subjects should chase and attempt to capture a virtual prey that was programmed to run away from them. A pink boundary signals to the subject that they will encounter the virtual predator and should prepare to escape (i.e., the Escape Condition). Note that we signaled the type of the next task since we wanted to make sure that participants know whether they should switch or stay at the timing of the Switch or Stay screen, which screen will be used as a time window of the MVPA analysis. These boundaries are either congruent with the previous task (in our example, hunt) or incongruent allowing us to examine the switch between offensive and defensive behaviors. Boundary colors, the color of the subject, and the color of the predator were counterbalanced across subjects.

Step 2. After the cue indicating either the Hunt or Escape conditions, a short ITI (0.5 to 1.5 s) was presented, after which the subject occasionally entered a pre-Hunt or pre-Escape condition (i.e., pre-encounter stage 0 to 10 s). This pre-encounter stage was introduced to examine preparatory behavior before the main task. For example, in animal studies, thigmotaxis is a pre-encounter stage behavior that occurs when animals anticipate the presence of a predator in the near future [58]. This behavioral index allows experimenters to test whether animals properly understand the experimental context. Similarly, we utilized thigmotaxis as a behavioral marker to indicate whether participants appropriately understood the experimental context (boundary colors). The pre-encounter stage was designed to occur with a 40% probability when the previous trial was terminated by the capture, resulting in 19.2% of trials having a pre-encounter stage. In the pre-encounter stage, the subject prepared for the predator or prey to enter the arena, allowing us to examine post-encounter threats at the neural and behavioral levels. Note that since the Switch/Stay screen appeared before the pre-encounter stage of the next trial, the presence of the pre-encounter stage did not affect the BOLD signal of the Switch or Stay screen which was used for the analysis of the hypothalamic switching signal in the MVPA and the IC analyses.

Step 3. In the main task stage (2 to 10 s), the subject encountered the predator (i.e., the Escape Condition) or the prey (i.e., the Hunt Condition). Subjects used an MRI-compatible joystick to escape from the virtual predator (in the Escape Condition) or hunt the virtual prey (in the Hunt Condition). Experimental task was terminated after random (2 to 10 s) or when the player captured the prey in the Hunt Condition or was captured by the predator in the Escape Condition. Successful hunt was defined as the capture of the prey before the termination of each trial while the Successful escape was defined as not being captured until the termination of each trial. The subject’s position was updated every frame (16 ms) and the speed of movement was fixed throughout the experiment (0.006 unit/frame; here 1 unit represents the height of the screen). If the subject was captured by the virtual predator in the Escape Condition or failed to capture the virtual prey in the Hunt Condition, there is a 14% chance of receiving an electric shock. We calibrated the intensity of the shock before the experiment such that every participant felt a moderate degree of uncomfortableness with the shock (a degree that they do not want to get shocked that is around 5 on a visual analog scale between 0 to 10). Thus, the contingencies were identical for Hunt and Escape conditions. An artificial agent was programmed to move every frame (16 ms), and its movement was based on the A-star algorithm [16,59], which is commonly used in video gaming. The algorithm aims to minimize the cost function of future positions by considering the player’s possible movements (30 positions equally spaced on a circle centered on the current position with a radius of speed). The cost function also increases with the distance from the center. In the Hunt Condition, the cost function of the computer agent’s future position was increased by the distance from the subject while in the Escape Condition, the cost function of the future position was decreased by the distance from the player. In both conditions, the cost function was also increased by the distance from the center [16]. Importantly, subjects’ success rates were tracked every 4 trials during the experiment, and the speed of the computer agent was calibrated continuously according to the subjects’ performance on each task to make the subjects’ success rate around 50%. Furthermore, in the escape task, the speed of the predator was irregular such that the computer agent made a boosting movement randomly, which is intended to induce dynamic escaping movement of subjects. This boosting promoted predictive, nonlinear escaping behavior in participants, rather than simple circling movements. When the predator overshot due to the boost, the optimal strategy was to move away from the predator’s expected trajectory.

Step 4. While subjects were playing one type of task (i.e., the Hunt task) or when the task is terminated by catching prey in the Hunt task or being caught by the predator in the Escape task, a screen that informs players about the next task (Switch/Stay screen) appear randomly (between 2~10 s). The subject will either be in the same condition (i.e., hunting the prey again; “stay” condition, 50% probability) or will switch (from hunting virtual prey to escaping from the virtual predator; “switch” condition, 50% probability). After the Switch/Stay screen, the position of the player and the computer agent remained the same unless the trial was terminated by capture (either the player captured the prey or the player was captured by the predator). In those cases (after capture), the player and the computer agent were respawned in a random location. Respawning locations of computer agents were sampled from the 2-D Gaussian distribution with a mean of (0, 0) (center), and the covariance matrix of an identity matrix having a magnitude of 1/4 radius of the field while the respawning locations of the player were sampled such that distance from the center follows uniform distribution having range of [0, radius]. Since the predator was more likely to be spawned close to the center, locating close to the boundary before the escape and locating close to the center was an optimal strategy in the pre-encounter stage. Based on performance (average success rate in all trials including both Hunt and Escape Conditions) during the task, participants earned a monetary reward between 8$ and 20$. Additionally, on a random 10% trial, subjects were asked to rate their confidence of success between 1 to 7 before starting each hunt or escape condition. For conciseness, confidence ratings were not used in this study.

The experimental paradigm for the online behavioral experiment (experiment 1) was very similar to the fMRI experiment except for some minor details including the use of the Keyboard instead of the Joystick which had a lower degree of freedom (for the details, see the S1 Text). Lastly, note that this task differs from the approach-avoidance task since the approach-avoidance task involves decision-making between approach and avoidance while our task explicitly cues the type of task without decision-making between hunt and escape.

### Experimental paradigm for the control task (in experiment 2)

To test whether the hypothalamus encodes only switching between survival behaviors such as escaping and hunting, or whether this region also encodes motor and attentional switching that is not related to survival, we designed the control task. Participants performed 4 runs of this control task after finishing the main experimental task. This task involves switching or staying (containing 60 Switch and 60 Stay each) between a task of collecting numbers from 1 to 4 in ascending order or collecting numbers from 4 to 1 in descending order. The success of the trial was defined as correctly collecting all numbers in the right order before the trial ended; otherwise, it was considered a failure.

Similar to the main experimental task, participants were asked to move their avatar to collect numbers in an appropriate order in each trial and the type of the task (ascending versus descending) was signaled by the boundary color (blue for ascending order, red for descending order; counterbalanced across subjects). The length of the Switch/Stay screen and the length of the main trial were the same as the experimental task. The position of the numbers was fixed throughout the trial.

We intended to design the control task that is as identical as possible to the experimental task in terms of the motor perspective while not including the survival goals (reward, loss, and shock) and the survival behaviors (escaping and hunting which is more natural, innate behavior than number collecting). Therefore, the control task was designed to induce a switching between 2 continuous behaviors that require motor planning, since continuous movement and motor planning are 2 key motor components of the experimental task. Also, there were 2 major differences between the experimental task and the control task. First, unlike in the experimental task, the control task did not include a survival “goal” to get a reward and to avoid shock. Participants did not get a reward after success nor got electrical shock after failure in each trial. Furthermore, the control task also did not include naturalistic survival actions such as pursuing and attacking prey and escaping from the predator which is an innate survival action that happens frequently in the naturalistic environment. Those survival behaviors have been preserved from low-level animals like reptiles to high-level animals such as humans and primates while collecting numbers in ascending/descending order is an artificial task having an abstract goal that requires an understanding of written numbers and converting it to an abstract goal that does not frequently happen in a naturalistic environment. Note that the number of trials in the control task was smaller than in the experimental task (41 trials × 4 sessions in the control task; 121 trials × 4 sessions in the experimental task). This was done to reduce total scanning time, which was already very long without control task (approximately 1.5 h × 2 days).

### Questionnaire

In both experiments (experiments 1 and 2), participants were asked to complete psychological surveys including state-trait inventory for cognitive and somatic anxiety-trait (STICSA-trait), behavioral approach system–behavioral inhibitory system questionnaire (BISBAS), perceived stress scale (PSS), aggression questionnaire (AGQ), and general medical history including psychiatric history to investigate a psychological trait associated with the switch. These questionnaires were not used in this study. They will be used in a separate study.

#### Behavioral analyses

We first examined the characteristics of escaping and hunting behavior. First, we tested how difference in preparation behavior before escaping and hunting behavior. Since the predator was more likely to occur near the center, optimal preparation behavior was to move close to the boundary (thigmotaxis) before escaping and to move close to the center before hunting. We compared the thigmotaxis between escaping and hunting. Here, thigmotaxis means the probability of the player residing close to the boundary (outside 50% area of the field). Furthermore, previous studies showed that escaping behavior entails a more unpredictable movement trajectory [26]. We defined 2 quantities to represent the unpredictability of the movement. The first quantity was the “movement variability” which was defined as an average change of movement direction (in degrees) per unit time (frame, 16 ms). The second quantity was a “linearity” representing the degree to which a player’s movement follows the extended virtual line between the computer agent and player. More specifically, a linear direction was defined as a movement direction toward the prey on the virtual line (vector from player to the prey, Fig 1D) in a hunting behavior while in the escaping behavior, it was defined as the movement direction away from the predator on the virtual line (vector from the predator to player, Fig 1D), which directions are thought to be most basic and intuitive, thus most expectable form of behaviors for both behaviors. Linearity was calculated by a dot product between linear movement direction and actual movement direction and we assumed that bigger linearity means more expectable behavior. We computed average linearity across every time point (unit is the frame) and trials for each condition (Hunt and Escape) and compared them.

### A generative model of the hunting and escaping behavior

In the behavioral analyses, we found a significant difference in behavioral patterns between hunting and escaping. We expected that an internal movement generation process of hunting and escaping might also be different. To test this hypothesis, we first constructed several generative models that can explain both hunting and escaping behavior and tested which model explains the behavior most accurately. Then, we tested a difference in the internal movement generation process between hunting and escaping by comparing model parameters in both conditions. Furthermore, we tested whether we could decode hunting and escaping states from the internal movement generation process by training the classifier with the parameters of the generative model. Significant decoding accuracy means that those 2 states are separable thus the internal movement generation process is highly condition-specific.

Similar to the recent study [16], our model assumes that an agent has an internal model of predator or prey that enables a prediction of their future position. By using this model, an agent can compute the value of each possible movement. For a Hunt task, the value was higher for the movement that makes an agent closer to the future position of the prey while in the escape task, the value was higher for the movement that makes an agent farther from the future position of the prey. Using this value function, an agent makes a movement decision.

More precisely, an agent made a prediction at time t for the next position of the computer agent (*y*_*pred*_(t)) based on its current position (y), velocity (y′), and acceleration (y”) as follows:

$$y_{pred}(t)=y(t)+y\prime (t)*\theta +y\prime \prime (t)*\frac{1}{2}\theta^{2}$$

Where parameter *θ* represents the time step size of the prediction.

Value function (*Q*(*x*(*t*), *y*(*t*), *a*(*t*)) of movement at time t (a(t)) was computed based on the Euclidean distance between an agent’s current position (*x*(*t*)) and the predicted position of *y*_*pred*_(t). Note that although the number of directions that participants can move using the Joystick was infinite in experiment 2, we discretized movement directions into 128 directions. Therefore, the number of possible movements was 129 (128 directions + 1 no movement). In experiment 1, the number of possible movements was 9 (8 directions using keyboard + 1 no movement).

The value function for movement in the hunt task was computed as below:

$$Q(x(t),y(t),a(t))={-d(x(t),y_{pred}(t))}^{2}$$

The value function for movement in the escape task was computed as below:

$$Q(x(t),y(t),a(t))={d(x(t),y_{pred}(t))}^{2}$$

Here, d(x,y) means an Euclidean distance between x and y. The value of each movement was submitted to a Softmax function to make a decision

$$p(a(t))=\frac{\tau *Q(x(t),y(t),a(t))}{\sum_{a\in A}exp(\tau *Q(x(t),y(t),a(t)))}$$

Here, τ determines the degree of deterministic goal-appropriate decision such that when τ is high, the player will make a task-appropriate movement with high probability (e.g., moving directly toward a predicted location of the prey in Hunt condition), while the τ is negative, a player will likely to make a task-inappropriate movement (e.g., moving away from the predicted location of the prey in Hunt condition). Finally, if the τ is close to 0, the randomness of the movement will increase since the player will make a movement irrelevant to the value function with high probability when τ is high. A is the set of all possible actions (129 actions in experiment 2; 9 actions in experiment 1).

In Model 1 (M1 in Fig 2C), a player does not make a prediction about the movement of the future position of the computer agent (*y*_*pre*d_(t) = y(t); no *θ* in this model) and makes decisions only based on the current position of that agent (Reactive model).

In Model 2 (M2 in Fig 2C), a player at time t makes a prediction about the position at time t+*θ* of the computer agent. This predictive computation and the movement decision were assumed to be made in every time step (which means planning and decision occur every frame (16 ms)). Velocity (y′(t)) and acceleration (y”(t)) at time t was computed as follows:

$$y^{\prime }(t)=y(t)-y(t-1)$$

$$y^{\prime \prime (t)}=y^{\prime (t)}-y^{\prime (t-1)}$$

In Model 3 (M3 in Fig 2C), we thought that the movement planning and decision might not occur every time step. More specifically, we hypothesized that there would be a time unit of computation (including planning and decision making) that varies between subjects and varies within subjects (e.g., influenced by context such as Hunt and Escape condition). In this model, movement planning and decision occur in a time scale of *θ*. Therefore, the computation of velocity (y′(t)) and acceleration (y”(t)) at time t also occurs in a time scale of *θ* such that:

$$y^{\prime }(t)=y(t)-y(t-\theta )$$

$$y^{\prime \prime (t)}=y\prime (t)-y\prime (t-\theta )$$

We assumed that the player will keep movement toward the same direction for time *θ* after making a movement decision at time t (thus the same movement until time t+*θ*). The probability of keeping the same direction for the duration of *θ* was parameterized as μ such that there is a probability 1-μ of making noisy, non-planned movement. We assumed that the parameters of the computational model vary across time, following a similar approach in the previous study [16]. Participants’ behavior in each trial was segmented into 500 ms segments (30-time points) with a step size of 250 ms by adapting the moving window approach. We fitted our model to these 500 ms segments separately using the “fmincon” function of MATLAB 2020b to get time courses of model parameters. This enables us to quantify the state of the internal movement generation process and investigate the effect of context (e.g., hunting versus escaping or switching versus staying) on this process. Additionally, the moving window of model fitting was started from the time point that the participant started their movement in each trial as we assumed that the movement generation process was started from that time point (thus the first few time points of every trial that participants did not make movement was excluded from model fitting). Finally, 3 models were compared using the Bayesian model selection procedure [60] based on the average Bayesian information criteria (BIC) of every model.

### Decoding task movement generation process

To test the difference between the internal movement generation process of hunting and escaping, we trained a classifier to decode each task state (hunting and escaping) based on the parameters of the computational model. If internal movement generation 2 states are different, decoding accuracy would be significantly above the chance level. We trained the support vector machine (SVM) classifier (“fitcsvm” function in Matlab was used; https://www.mathworks.com/) to decode the Hunt or Escape condition based on 3 parameters (*θ*, *τ*,μ) of Model 3 (winning model). Model parameters were averaged within each trial, thus we had 1 representative parameter set for each trial (161 parameter sets in experiment 1; 484 parameter sets in experiment 2) and decoding accuracy was computed by leave-one-trial-out cross-validation.

### Defining condition-specificity of movement generation state

We found that the task state can be reliably decoded from the internal movement generation state with high accuracy (approximately 90%), meaning that the movement generation state is highly specific to both tasks (hunting and escaping task) such that their movement generation parameter setting is different.

We expected that a higher Condition-specific state that is appropriate to the current task (e.g., decoder output indicates a 95% probability of hunting in the Hunt condition or indicates a 95% probability of escaping in the Escape condition) would be associated with a more optimal internal movement generation process. If so, a (task-appropriate) higher condition-specific movement generation state would predict the higher probability of success in each trial. To test this hypothesis, we performed mixed-effect logistic regression analyses to explain success in each trial using average task specificity (C_P_). C_P_ of each time window (500 ms segment used for model fitting) was defined as the task-appropriate decoding probability such that if the probability of hunting state is P (from the decoder output) and the probability of escaping is 1-P, C_P_ is P if the current task is hunting while the C_P_ is 1-P if the current task is escaping. The average C_P_ for each trial was calculated by averaging C_P_ for every time window in each trial. Note that the output of the SVM classifier was transformed to the probabilistic output (between 0 and 1) by applying the Matlab function “FitPosterior.” Age and sex were entered as covariates and the subject, and run number (only in experiment 2) were used as the random-effect variables. Covariate and random effect variables will be the same in all following regression analyses.

### Coordination of condition-specific movement generation state after switching

We next investigated how the transition of the internal movement generation process is coordinated after switching (e.g., transition of hunting behavior to escaping). The transition of the movement generation process after the switch was quantified by subtracting 1-C_P_ of the previous trial’s last moment (1- C_P_ of the final segment just before the Switch/Stay occurs) from the initial C_P_ of the current trial. For example, if the decoding probability of the final segment of the previous trial (e.g., hunting trial) was 24% escaping and 76% hunting and the initial decoding probability of the current trial (e.g., escaping) is 60% escaping and 40% hunting, the amount of transition is 36% since the 1-C_p_ of the previous hunting trial is 24 and the C_P_ of the current escaping trial is 60. We then tested if this transition is larger than 0 and larger than the transition after stay.

Then, we expected that the switching process would involve the suppression of the previous Condition-specific state. More specifically, if the switching process involves the suppression of the previous state, we expected that the transition from the previous state to the current state would be more difficult if the previous state was highly condition-specific (e.g., if the state of previous trial was 90% escape, transition to hunting state would be more difficult compared to when previous state was 60% escape) since the suppression loading would be high compared to when the previous task is not condition-specific (e.g., C_p_ is around 50%). This was tested using mixed-effect linear regression predicting the initial C_P_ of the current trial using the final C_P_ of the previous trial as a predictor variable in switched trials. Also, we tested the time to achieve substantial task specificity (time to achieve C_P_ = 0.6 after the start of the trial; C_P_ = 0.5 means that the current movement generation state is completely nonspecific neither to hunting nor escaping).

### FMRI data acquisition

fMRI data was collected using a 3T Prisma scanner with a 32-channel head coil in the Caltech Brain Imaging Center. BOLD contrast images were acquired using a single-shot, multiband T2*-weighted echo planar imaging sequence with the following parameters: TR/TE = 500/30 ms, Flip Angle = 60°, 30 slices, slice angulation = −20°, multiband acceleration = 6, slice thickness = 2.0 mm, FOV = 192 mm × 192 mm). Note that based on a previous study showing significant benefits of shortening repetition time (TR) in MVPA analyses [61], we used short-TR of 500 ms at the cost of reduced coverage which did not include whole-brain.

We thoroughly selected the coverage of the MR signal acquisition to include the hypothalamus, which is the most important region of interest in our study. However, we failed to have a signal of this region in one subject among 22 subjects who underwent scanning possibly due to excessive movement. Therefore, we excluded this subject from all analyses, resulting in 21 subjects being included in the analyses. Coverage also included the thalamus, hippocampus, amygdala, bilateral dorsolateral prefrontal cortices, and anterior cingulate cortex. However, periaqueductal gray was not included in 4 subjects among 21 included subjects. Therefore, analyses for periaqueductal gray were done in 17 subjects. Anatomical reference imaging employed 0.9 mm isotropic resolution 3D T1w MEMP-RAGE (TR/TI/TE = 2,550/1,150/1.3, FOV = 230 m × 230 mm) and 3D T2w SPACE sequences (TR/TE = 3,200/564 ms, FOV = 230 mm × 230 mm). Participants viewed the screen via a mirror mounted on the head coil. Electric stimulation was delivered using a BIOPAC STM100C. Participants used Joystick (NAtA Technology; https://natatech.com/; Product number: FOJ-2B-10B) inside the MRI scanner which enabled them to move freely toward every direction (inside the experimental field) at a fixed speed.

### fMRI preprocessing

Participants’ data were preprocessed using fMRIprep [62] (version 20.2.3 stable). Briefly, the T1w image was corrected for intensity non-uniformity and skull-stripped. Spatial normalization to the ICBM 152 Nonlinear Asymmetrical template version 2009c was performed through nonlinear registration using brain-extracted versions of both T1w volume and template with ANTs. Brain tissue segmentation of cerebrospinal fluid (CSF), white matter (WM), and gray matter (GM) was performed on the brain extract. Functional data were motion corrected with the mcflirt function of FSL [63] and was followed by co-registration to the corresponding T1w using boundary-based registration with 6 degrees of freedom. Motion correcting transformations, BOLD-to-T1w transformation, and T1w-to-template (MNI) warp were concatenated and applied in a single step using antsApplyTransforms from ANTs [64] using Lanczos interpolation. Physiological noise regressors were extracted by applying CompCor. Principal components were estimated for the 2 CompCor variants: temporal (tCompCor) and anatomical (aCompCor). A mask to exclude signals with cortical origin was obtained by eroding the brain mask, ensuring it only contained subcortical structures. Six tCompCor components were then calculated including only the top 5% variable voxels within that subcortical mask. For aCompCor, 6 components were calculated within the intersection of the subcortical mask, and the union of CSF and WM masks was calculated in T1w space, after their projection to the native space of each functional run. Framewise displacement was calculated for each functional run using the implementation of Nipype [65].

### MVPA analyses

MVPA analyses to find regions encoding switch or stay information were performed following the COSMOMVPA toolbox pipeline [32] both in regions of interest (ROIs) level and in all-voxel level (all voxels covered in our experiments) using searchlight methods. First, a single beta image for every trial except the first trials of each run at the switch or stay screen was estimated by Least-squares-single-2 (LSS-2) approach [66], which models event-related blood oxygenation level-dependent (BOLD) signal change, controlling for signal change due to all other trials and motion artifact. This step was done using the SPM12 software [67]. Note that the beta image for the first trial of each run was not constructed since the first Switch/Stay screen did not contain any Switch or Stay information (as there is no previous task), resulting in 480 single-trial beta images. LSS model of each single trial contains the first regressor as a boxcar function placed at the timing of the Switch/Stay screen (from onset to offset of Switch/Stay screen) of each trial (regressor of interest) and other regressors of non-interests including other trials and confound regressors. We selected 20 confound regressors among all confound regressors derived from fMRIPrep following previous recommendation [68] which included 6 motion parameters, Framewise displacement, DVARS (D, temporal derivative of time courses; VARS, RMS variance over voxels), 6 anatomical component-based noise correction (CompCor) components, and 3 cosine drift terms. Furthermore, we included CSF, WM, and global signal time series regressors to exclude their confounding effect, especially on the hypothalamus. Note that there has been an increasing interest in signals that have been previously interpreted as noise, such as low-frequency fluctuations in BOLD signal [69] and there is also an ongoing debate about whether CSF/WM signal contains a neural signal [70], which has been unclear so far. However, both CSF/WM signals have been conventionally removed in 63% of resting-state fMRI studies [71], and the removal of these signals improved the spatial specificity in BOLD-based seed maps [70]. Furthermore, the importance of removing the CSF signal becomes more important when it comes to studying the hypothalamus since it is close to third ventricle thus almost all recent fMRI studies of the hypothalamus regressed out CSF [20,72–74]. Therefore, we decide to regress out CSF/WM signal in this study to acquire a better hypothalamic BOLD signal.

We constructed a set of beta images for every trial (480 beta images) such that in one set of beta images, the switch time was early switch time while in another set, the switch time was late switch time. All MVPA analyses used the linear discriminant analyses (LDA) classifier of the COSMOMVPA toolbox with a leave-one-run cross-validation procedure. LDA classifier was the best classifier in decoding naturalistic stimuli in the previous study [75] and is computationally efficient and thus faster than most classifiers, such as SVM classifier, with comparable [76], or better classification performance [77] and also it has higher noise-resistance [78]. Choosing this classifier in MVPA analyses is especially beneficial for computationally demanding searchlight analyses.

### ROI selection and segmentation for MVPA analyses

Using 2 sets of beta images (early switch beta images and late switch beta images), we first performed MVPA analyses in 9 ROIs including the hypothalamus and other 8 regions that were previously known to be involved in task switching or encoding of survival behavior (hunting or escaping) or both in animal and human studies. The periaqueductal gray (PAG) has been involved in the coordination of switching between predation and evasion in connection with the hypothalamus [13] and the amygdala (AMY) has been known for regulating approach-avoidance in connection with the hypothalamus [55]. The anterior cingulate cortex (ACC; note that we did not separate dorsal/ventral ACC) and the ventromedial prefrontal cortex (VMPFC) have been associated with both task switching as well as the coordination of the survival behavior [5,6,16,49,79]. The thalamus (T) and left and right dorsolateral prefrontal cortices (DLPFC_L and DLPFC_R, respectively) have been associated with general task switching in previous studies [27]. Finally, the hippocampus has been known for integrating contextual information for the coordination of survival behaviors [29].

Since the hypothalamus is small and difficult to define using existing atlases, especially due to the small contrast difference with surrounding tissues, we applied deep-learning-based automated segmentation of the hypothalamus to an individual T1 image [19]. Classical approaches to hypothalamic segmentation have been dominated by atlas-based segmentation and manual segmentation. However, the accuracy of atlas-based segmentation is limited due to the small size of the hypothalamus, lack of image contrast in MRI, and individual variability in its shape [19]. Furthermore, the choice of atlas introduces bias in the results. Manual segmentation of the hypothalamus is also challenging due to its small size, low contrast, scalability, and reproducibility issues, possibly related to high inter-rater variability, as well as the time and effort required for manual segmentation [19].

The deep-learning-based segmentation approach we utilized in this study trains a deep convolutional neural network to segment the hypothalamus and its subnuclei. This approach utilizes data augmentation techniques to make segmentation more resilient against variations such as subject positioning and imaging artifacts [19]. This algorithm showed superior accuracy to conventional atlas-based segmentation and manual segmentation with fast implementation time [19]. Therefore, we chose to use this deep-learning-based segmentation approach rather than other conventional segmentation methods. Applying this algorithm enabled the identification of individual-specific hypothalamus using individual T1 images, as well as the identification of subunits of the hypothalamus including the anterior-inferior, anterior-superior, inferior-tuberal, superior-tuberal, and posterior subunits in our T1 MRI data. Among these subunits, our hypothalamus ROI was defined by concatenating subunits that contain lateral hypothalamus and ventromedial hypothalamus that were known to be involved in survival behavior (hunting and escape) in previous studies [12,13]. Those subunits were the posterior, inferior tubular, and superior tubular subunits.

Similar to the hypothalamus, thalamus ROI was defined to include the dorsomedial thalamus which was consistently associated with task switching in previous literature [27,80] using Oxford thalamic connectivity atlas [81]. The hippocampus and amygdala were defined using Harvard-Oxford cortical and subcortical structural atlases [82]. Finally, periaqueductal gray, dorsolateral prefrontal cortices, anterior cingulate cortex, and ventromedial prefrontal cortex were defined using Neurosynth meta-analyses (https://neurosynth.org/; terms “periaqueductal,” “dorsolateral prefrontal,” “anterior cingulate,” “ventromedial prefrontal” were used respectively with an association test). MVPA was performed in those 9 ROIs and *p*-values were FDR-corrected for 9 ROIs.

### Searchlight MVPA analyses

Searchlight analysis was performed over all covered voxels to find out regions encoding switch/stay information which might possibly include regions that were not covered by the ROI analyses. LDA classifier was trained across the 6-mm sphere to discriminate switch or stay at the time of late switch time. *P*-values of every voxel were corrected by threshold-free cluster enhancement (TFCE) methods [83].

### MVPA analyses for the control task

The same MVPA analyses we performed in an experimental task (in ROI-level and all-voxel level) were also done for the control task.

### Identifying network encoding Switch/Stay information

We measured multi-voxel functional connectivity called informational connectivity [23–25,34] between every pair of 9 ROIs and detected a brain network within those interactions using NBS [84]. IC measures a covariation of multi-voxel patterns encoding Switch/Stay information between 2 regions [24]. IC was computed by using the Informational Connectivity Toolbox (https://lrdc.pitt.edu/coutanche/informationalconnectivity)), which measured trial-by-trial covariation of distance between multi-voxel patterns encoding Switch/Stay information from the separating hyperplane [23,24] between every pair of 9 ROIs we used in previous MVPA analysis. After computing IC between every ROI, we applied NBS to identify a network encoding Switch/Stay information. The threshold for NBS was *p* < 0.05. To further define character characteristics of this network, such as finding hubs in the network, we computed node betweenness centrality (BC) [85] of each node that represents the fraction of all shortest paths that contain a specific node. Therefore, a node with higher BC means stronger influence over the flow of information between other nodes.

## Supporting information

S1 Fig Computational modeling results. **(A) Bayesian model selection results.** In both experiments, Predictive planning with a variable time scale (M3) was the winning model. **(B) Parameter distribution.** In the escaping behavior, compared to the hunting behavior, *θ*, the time step of computation was shorter (Escape vs. Hunt: 10.9 vs. 15.6 in experiment 1; 11.4 vs. 15.5 in experiment 2; all *p* < 0.001 in mixed-effect regression) and the *τ* was lower (Escape vs. Hunt: 29.3 vs. 383.8 in experiment 1; 49.6 vs. 322.0 in experiment 2; all *p* < 0.001 001 in mixed-effect regression), meaning the movement decision was less deterministic toward an ideally optimal linear direction (direction minimizes cost function in the generative model). However, the result regarding mu was inconsistent between the experiments such that in experiment 1, the probability of consistent movement between consecutive time steps was higher in hunting behavior while in experiment 2; this probability was lower in hunting behavior (Escape vs. Hunt: 0.95 vs. 0.95 in experiment 1; 0.74 vs. 0.68 in experiment 2; all *p* < 0.001 001 in mixed-effect regression). This could be attributed to the modality of movement the participant used (experiment 1: Keyboard; experiment 2: Joystick) where the Joystick is more sensitive to noisy movement that is not caused by movement computation (e.g., small twitching or movement affected by external factors). **(C) Time course of the C**_**p**_. We plotted the time course of the C_p_ from the start of the trial to 8 s for both online and fMRI experiments. This figure shows that C_p_ is close to 0.5 (around 0.6) at the start of the trial but rapidly increases until approximately 2 s to reach the maximum C_p_, showing that participants successfully learn the task of that trial quite quickly. Then, C_p_ fluctuates between decreasing and increasing.
(TIF)

S2 Fig Searchlight analyses results. **(A) Searchlight analyses of the experimental task.** Searchlight analyses of the main experimental task to decode Switch/Stay showed perigenual ACC, which we did not find in our ROI-based MVPA and bilateral visual cortex encodes Switch/Stay information. Note that we did not discriminate between dorsal/ventral ACC in our ROI definition, since the focus of this study was the hypothalamus. However, this could be one possible reason that we did not find significant encoding of Switch/Stay in ACC in ROI-based MVPA analyses. **(B) Searchlight analyses of the control task.** Searchlight showed that the small area within the left DLPFC significantly encodes to switch/stay.
(TIF)

S3 Fig IC analysis of the control task. A network that encodes switching in the control task is composed of the thalamus, hippocampus, amygdala, ACC, and bilateral DLPFCs. However, unlike the survival behavior switching network, this network did not include the hypothalamus and the VMPFC.
(TIF)

S1 Table Initial values and ranges of model parameter estimation (M3). (DOCX)

S1 Text Experimental paradigm for the online behavioral experiment (experiment 1). (DOCX)

S2 Text Comparison between the main experimental task and the control task. (DOCX)

S3 Text Identifying different roles of amygdala subnuclei on switching. (DOCX)

S4 Text Potential confounders of the hypothalamic switching signal. (DOCX)

## Abbreviations

ACC: anterior cingulate cortex
AGQ: aggression questionnaire
BC: betweenness centrality
BIC: Bayesian information criteria
BLA: basolateral amygdala
BOLD: blood oxygenation level-dependent
CMA: centromedial amygdala
CNN: convolutional neural network
CSF: cerebral spinal fluid
GM: gray matter
IC: informational connectivity
LDA: linear discriminant analysis
MVPA: multi-voxel pattern analyses
MVPSS: multi-voxel pattern strength of switching
NBS: network-based statistics
PAG: periaqueductal gray
PSS: perceived stress scale
ROI: region of interest
SVM: support vector machine
TFCE: threshold-free cluster enhancement
VMPFC: ventromedial prefrontal cortex
WM: white matter
