# Supplementary figures and images for: The human hypothalamus coordinates switching between different survival actions
Source: PLoS Biol. 2024 Jun 28;22(6):e3002624. doi: 10.1371/journal.pbio.3002624 (PMC11213486; doi:10.1371/journal.pbio.3002624)

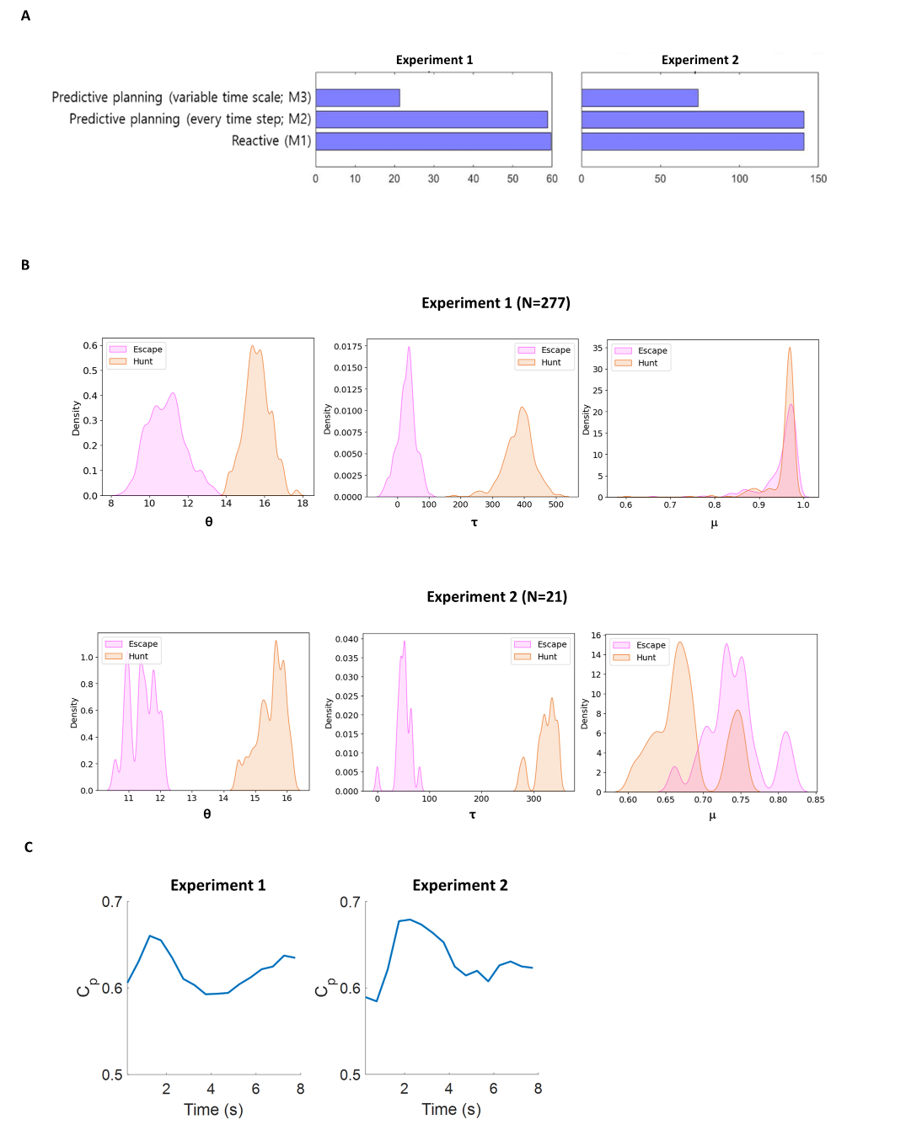

Supplement: S1 Fig — (A) Bayesian model selection results. In both experiments, Predictive planning with a variable time scale (M3) was the winning model. (B) Parameter distribution. In the escaping behavior, compared to the hunting behavior, θ, the time step of computation was shorter (Escape vs. Hunt: 10.9 vs. 15.6 in experiment 1; 11.4 vs. 15.5 in experiment 2; all p < 0.001 in mixed-effect regression) and the τ was lower (Escape vs. Hunt: 29.3 vs. 383.8 in experiment 1; 49.6 vs. 322.0 in experiment 2; all p < 0.001 001 in mixed-effect regression), meaning the movement decision was less deterministic toward an ideally optimal linear direction (direction minimizes cost function in the generative model). However, the result regarding mu was inconsistent between the experiments such that in experiment 1, the probability of consistent movement between consecutive time steps was higher in hunting behavior while in experiment 2; this probability was lower in hunting behavior (Escape vs. Hunt: 0.95 vs. 0.95 in experiment 1; 0.74 vs. 0.68 in experiment 2; all p < 0.001 001 in mixed-effect regression). This could be attributed to the modality of movement the participant used (experiment 1: Keyboard; experiment 2: Joystick) where the Joystick is more sensitive to noisy movement that is not caused by movement computation (e.g., small twitching or movement affected by external factors). (C) Time course of the Cp. We plotted the time course of the Cp from the start of the trial to 8 s for both online and fMRI experiments. This figure shows that Cp is close to 0.5 (around 0.6) at the start of the trial but rapidly increases until approximately 2 s to reach the maximum Cp, showing that participants successfully learn the task of that trial quite quickly. Then, Cp fluctuates between decreasing and increasing. (TIF) [file pbio.3002624.s001.tif]

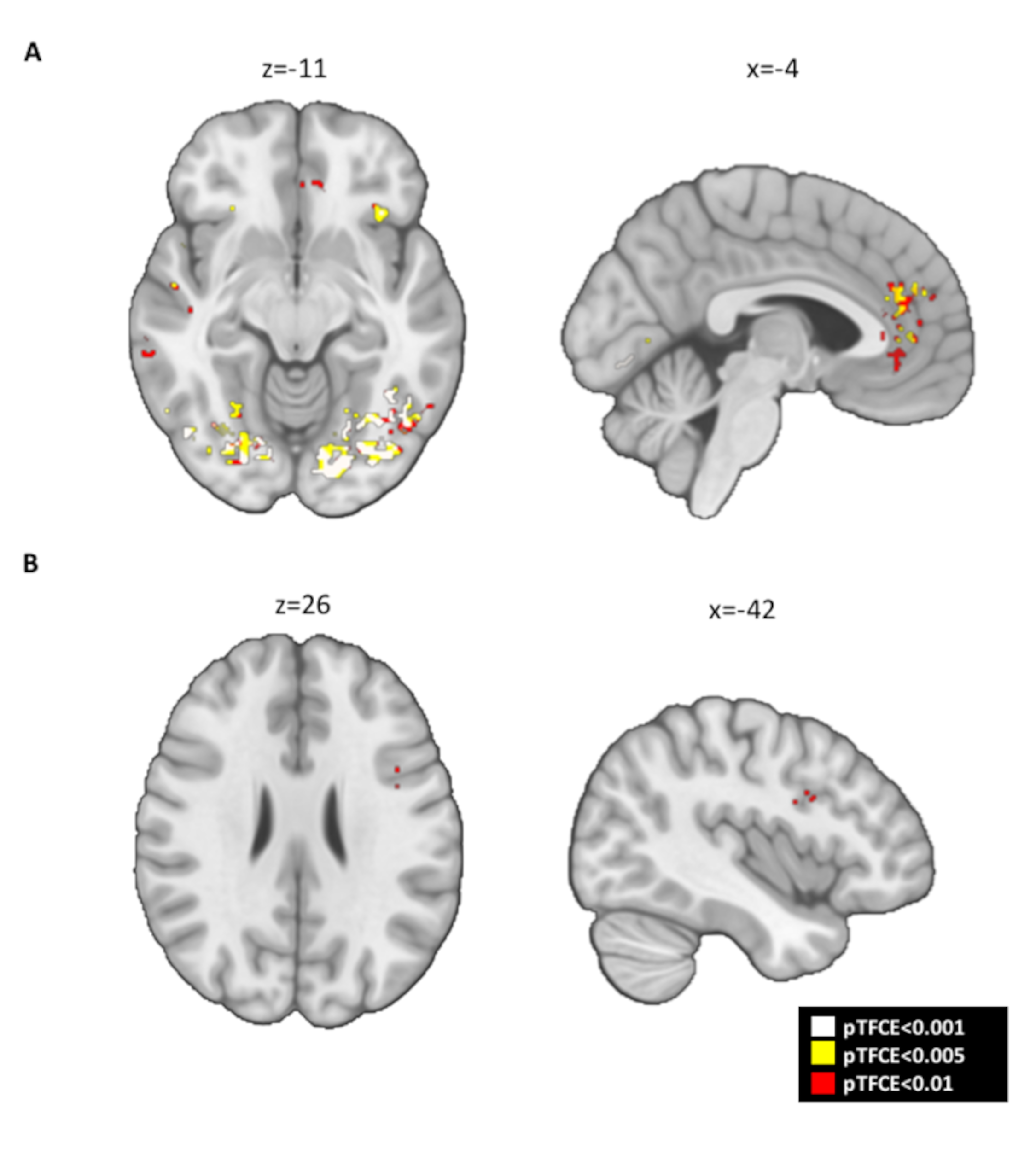

Supplement: S2 Fig — (A) Searchlight analyses of the experimental task. Searchlight analyses of the main experimental task to decode Switch/Stay showed perigenual ACC, which we did not find in our ROI-based MVPA and bilateral visual cortex encodes Switch/Stay information. Note that we did not discriminate between dorsal/ventral ACC in our ROI definition, since the focus of this study was the hypothalamus. However, this could be one possible reason that we did not find significant encoding of Switch/Stay in ACC in ROI-based MVPA analyses. (B) Searchlight analyses of the control task. Searchlight showed that the small area within the left DLPFC significantly encodes to switch/stay. (TIF) [file pbio.3002624.s002.tif]

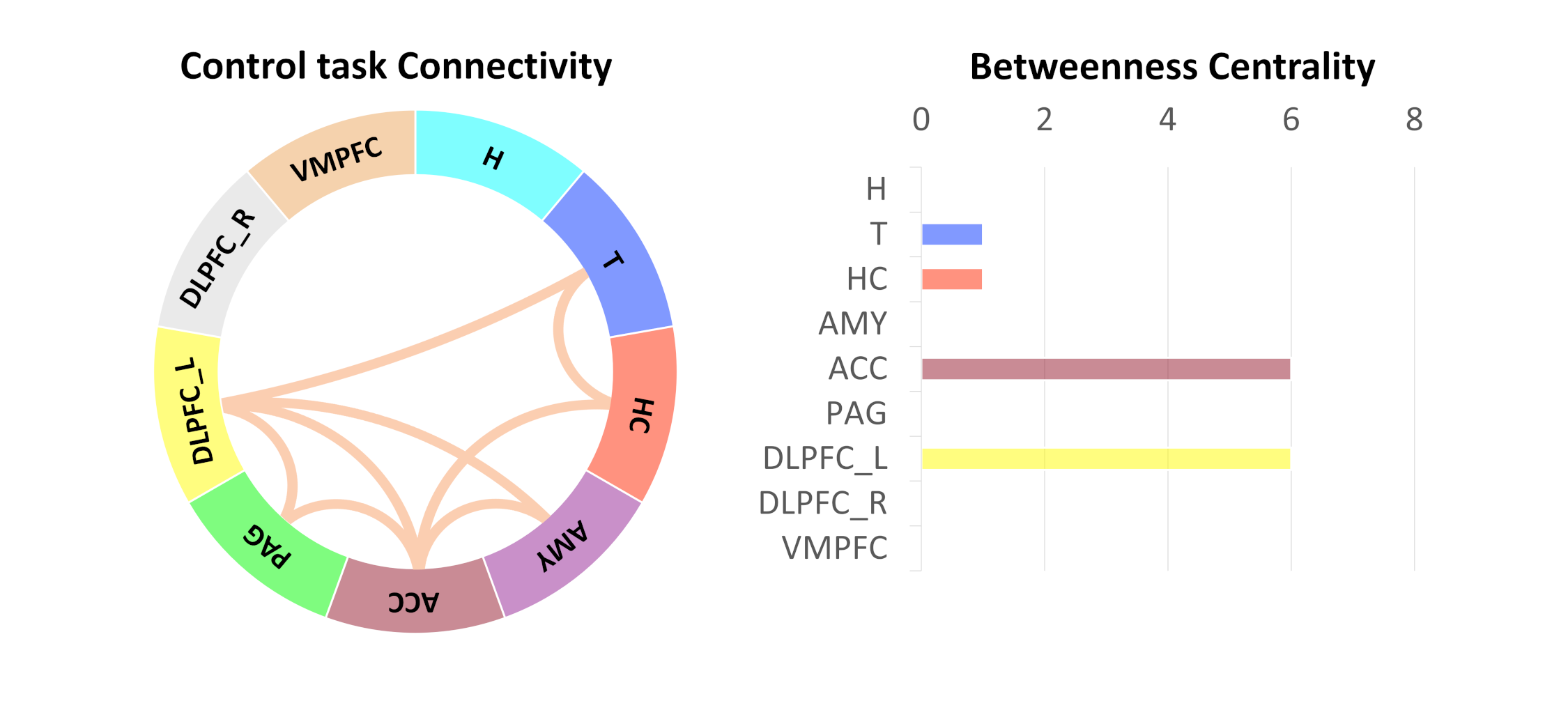

Supplement: S3 Fig — A network that encodes switching in the control task is composed of the thalamus, hippocampus, amygdala, ACC, and bilateral DLPFCs. However, unlike the survival behavior switching network, this network did not include the hypothalamus and the VMPFC. (TIF) [file pbio.3002624.s003.tif]
