# Supplementary material for: The human hypothalamus coordinates switching between different survival actions
Source: PLoS Biol. 2024 Jun 28;22(6):e3002624. doi: 10.1371/journal.pbio.3002624 (PMC11213486; doi:10.1371/journal.pbio.3002624)
Supplement: S1 Table — (DOCX) [file pbio.3002624.s004.docx]

**S1 Table**. Initial values and ranges of model parameter estimation (M3)

| Parameter | Initial values | | |
| --- | --- | --- | --- |
|  | Lower bound | Mean | Upper bound |
| $\theta$ | 0 | 5 | 30 |
| $\tau$ | -1000 | 1 | 1000 |
|  | 0 | 0 | 1 |

*The parameter settings were the same in other models (Model 1 does not have $\theta$ $,$and μ; Model 2 does not have θ)
