# Supplementary material for: The human hypothalamus coordinates switching between different survival actions
Source: PLoS Biol. 2024 Jun 28;22(6):e3002624. doi: 10.1371/journal.pbio.3002624 (PMC11213486; doi:10.1371/journal.pbio.3002624)
Supplement: S1 Text — (DOCX) [file pbio.3002624.s005.docx]

**S1 Text. Experimental paradigm for the online behavioral experiment (experiment 1)**

The task for the online experiment was the same as the fMRI experiment except that people used a Keyboard instead of the Joystick which had a lower degree of freedom (8 directions). Split across 161 trials, subjects were asked to either hunt the virtual prey or escape from the virtual predator. In the hunting condition (signaled by the green boundary), players had to chase and catch the computer agent while in the escape task (signaled by the red boundary), players had to escape from the computer agent. Subjects used the arrow keys of the keyboard to move which allows 8 movement directions including four diagonal movements by the combination of arrow keys. The screen was updated every 1/60 second (every participant was asked to use a 60Hz monitor setting and those who did not use this setting were excluded). Participants’ success rate was tracked for every four trials during the experiment and the speed of the computer agent was calibrated continuously (+15% or -13%) according to participants’ performance on each task to make participants’ success rate around 50%. Furthermore, in the escape task, the speed of the computer agent (predator) was irregular such that this computer agent made boosting movement randomly, which manipulation was intended to induce dynamic escaping movement of participants. Participants received one point for catching virtual prey or escaping from the virtual predator. On the other hand, they lost one point by getting caught or by failing to catch the prey. Note that we equated feedback in hunt and escape in Expt. 1 and Expt. 2 to avoid unnecessary confounding by involving different types of feedback in two conditions (e.g., reward/non-reward in the hunt condition and loss + potential shock /non-loss + omission of shock in escape condition).

Participants received a bonus according to the total amount of points. There was a “switch or stay screen” in the middle of each task showing the task type of the next trial. The type of task they are playing (e.g., hunt) could change suddenly into another type of task (escape) after this screen (‘switch’ condition, 50% probability) or the participants could play the same type of task after this screen (‘stay’ condition, 50% probability). This screen appeared randomly between 2s to 14s and lasted for 2s. There was also a period called the pre-encounter period before the hunt and escape task. During the pre-encounter period, players were able to move freely without a computer agent to prepare for the upcoming task. the length of the pre-encounter period varied randomly between 2s to 14s and this pre-encounter period appeared only after players were getting caught by the computer agent in the escape task or they successfully caught the prey in the hunt task. At the end of the pre-encounter period, a computer agent appeared from the random location around the center of the circle (following a 2-D Gaussian distribution whose mean is (0, 0) (center) and covariance matrix is an identity matrix having a magnitude of 1/4 radius). Participants were also asked to rate their confidence of success between 1 to 7 before starting each hunt or escape task in 20% of trials. Finally, before starting an experiment, participants were asked to escape from a simple maze within 30 seconds to test their basic skills to control the keyboard. Those who failed 4 times were not allowed to participate in the experiment.
