# Supplementary material for: The human hypothalamus coordinates switching between different survival actions
Source: PLoS Biol. 2024 Jun 28;22(6):e3002624. doi: 10.1371/journal.pbio.3002624 (PMC11213486; doi:10.1371/journal.pbio.3002624)
Supplement: S2 Text — (DOCX) [file pbio.3002624.s006.docx]

**S2 Text. Comparison between the main experimental task and the control task**

**MVPA of the Main task with 15 subjects who completed the control task**

In the main manuscript, we showed that the hypothalamus, hippocampus, and PAG encode survival switching but not non-survival switching in the control task. This could have been confounded by 1) the smaller sample size (N=21 in the experimental task vs 15 in the control task) and 2) smaller trial numbers in the control task. Suppose the smaller sample size confounded the results that H, HC, and PAG are involved in encoding switching in the Main task but not in the control task. In that case, the MVPA results in the Main task should disappear if we only involve 15 subjects who completed both the main and control tasks in the ROI-based MVPA analyses. To test this possibility, we performed MVPA on these three regions in 15 subjects who completed the control task. Hypothalamus was still marginally significant (pFDR = 0.052; FDR-corrected for three regions) and HC and PAG were still significant (pFDR = 0.008, 0.006 respectively) while none of these regions were far from significant in the control task (pFDR =0.5183, 0.3723, 0.3723 for hypothalamus, HC, PAG), showing that H, HC, and PAG is still encoding the survival switching in this smaller population while not encoding non-survival switching. However, the effect size was decreased in H and PAG (in terms of Cohen’s d; 0.543->0.449 in the hypothalamus, 0.739->0.636 in PAG) which could reflect the effect of smaller sample size in MVPA signal (HC signal was increased from 0.533 to 0.581).

**IC analysis of the control task**

To identify how the network encoding switching in the control task differs from the network encoding switching in the Main task, we ran an IC analysis between nine ROIs in the control task. We found a network that encodes switching in the control task composed of the thalamus, hippocampus, amygdala, ACC, and bilateral DLPFCs. Note that all these regions were also part of the survival behavior switching network. However, unlike the survival behavior switching network, this network did not include the hypothalamus and the VMPFC. In other words, the hypothalamus was not connected to any of the 8 ROIs to encode the Switching in the control task, including the hypothalamic-amygdala connection. Overall, the survival behavior switching network was more densely connected than the Switching Network of the control task. Interestingly, similar to the survival behavior switching network, ACC was the hub region of switching in the control task, suggesting the ACC’s role in the general switching process. The role of DLPFC_L was more prominent in the control task network than in the Main task (the region with the highest betweenness centrality along with ACC). To evaluate the possibility that the difference in the network between the control task and the Main task came from the different number of subjects involved in each task, we re-ran the Informational Connectivity analyses on the Main task with the subjects who completed the control task (N=15). Results were similar to the one that was performed on whole subjects (N=21), reproducing the survival behavior switching network including eight ROIs with ACC as a hub, and hypothalamus connected to ACC, PAG. However, in these N=15 subjects, the hypothalamus was not directly connected to an Amygdala but was connected indirectly through other ROIs such as the thalamus, ACC, and PAG.

VMPFC.
