# Supplementary material for: The human hypothalamus coordinates switching between different survival actions
Source: PLoS Biol. 2024 Jun 28;22(6):e3002624. doi: 10.1371/journal.pbio.3002624 (PMC11213486; doi:10.1371/journal.pbio.3002624)
Supplement: S3 Text — (DOCX) [file pbio.3002624.s007.docx]

**S3 Text**

**Identifying different roles of amygdala subnuclei on switching**

We showed that amygdala did not encode the switching nor predict optimal coordination after the switching. However, there is still a possibility that some specific amygdala nuclei (e.g., basolateral amygdala (BLA) or centromedial amygdala (CMA) might be more involved in the switching since there role has been known to be different ^1^. To address this possibility, we first tested decoding accuracy of the switching separately in the BLA and CMA. BLA and CMA were defined using the Julich histological atlas ^2^. Neither of these nuclei encoded switching (all p>0.4). Also, their MVPSS did not predict optimal movement coordination after switching (all p>0.2). Therefore, similar to the whole amygdala analyses, these nuclei might not be directly related to an encoding of the switch itself nor the movement coordination after switching. Finally, we tested which one of two nuclei have more active role in coordination of the optimal movement after switching in conjunction with the hypothalamus. To test this possibility, we tested which synchronization, between the hypothalamus-BLA synchronization and the hypothalamus-CMA synchronization, predicts an optimal movement coordination after the switching. Results showed that among those two synchronizations, only the hypothalamus-CMA synchronization (beta=0.02, t=1.99, p=0.047), but not the hypothalamus-BLA synchronization (p=0.341) predicts an optimal movement coordination.

This is consistent with animal studies consistently showing that central amygdala to hypothalamus pathway is associated with both an aggressive behavior ^3^ and an avoidance behavior ^4^. Furthermore, recent studies suggested that the central amygdala gathers information on sensory and higher-order processes from various cortical and subcortical regions ^5^ and BLA is one of the input region to the central amygdala ^1,5^. Based on these studies, we suspect that the central amygdala might be a region that conveys an integrated sensory information to the hypothalamus while BLA could be an upstream region that provides one of the contextual information to the central amygdala. Since this is just our speculation based on previous studies, inspired by your comment, any further feedback from you on this discussion would be appreciated.

**References**

1. Terburg, D., Scheggia, D., Rio, R.T. del, Klumpers, F., Ciobanu, A.C., Morgan, B., Montoya, E.R., Bos, P.A., Giobellina, G., Burg, E.H. van den, et al. (2018). The Basolateral Amygdala Is Essential for Rapid Escape: A Human and Rodent Study. Cell *175*, 723-735.e16. 10.1016/j.cell.2018.09.028.

2. Amunts, K., Kedo, O., Kindler, M., Pieperhoff, P., Mohlberg, H., Shah, N.J., Habel, U., Schneider, F., and Zilles, K. (2005). Cytoarchitectonic mapping of the human amygdala, hippocampal region and entorhinal cortex: intersubject variability and probability maps. Anat Embryol (Berl) *210*, 343–352. 10.1007/s00429-005-0025-5.

3. Haller, J. (2018). The role of central and medial amygdala in normal and abnormal aggression: A review of classical approaches. Neuroscience & Biobehavioral Reviews *85*, 34–43. 10.1016/j.neubiorev.2017.09.017.

4. Weera, M.M., Shackett, R.S., Kramer, H.M., Middleton, J.W., and Gilpin, N.W. (2021). Central Amygdala Projections to Lateral Hypothalamus Mediate Avoidance Behavior in Rats. J. Neurosci. *41*, 61–72. 10.1523/JNEUROSCI.0236-20.2020.

5. Fadok, J.P., Markovic, M., Tovote, P., and Lüthi, A. (2018). New perspectives on central amygdala function. Current Opinion in Neurobiology *49*, 141–147. 10.1016/j.conb.2018.02.009.
