# Supplementary material for: The human hypothalamus coordinates switching between different survival actions
Source: PLoS Biol. 2024 Jun 28;22(6):e3002624. doi: 10.1371/journal.pbio.3002624 (PMC11213486; doi:10.1371/journal.pbio.3002624)
Supplement: S4 Text — (DOCX) [file pbio.3002624.s008.docx]

**S4 Text. Potential confounders of the hypothalamic switching signal**

**Effect of emotional valence and arousal in the hypothalamic switching signal**

Since the hypothalamus has been associated with the processing of emotion and arousal ^1^, the hypothalamic involvement in switching found in our study might be related to arousal or emotional valence. However, first, because we concatenated both Switching from hunt to escape and switching from escape to hunt, even if we assume that the level of arousal or some emotional component is different between hunt and escape, these differences should have been canceled out. This means that the involvement of the hypothalamus in switching found in our study is less likely to be influenced by the difference in emotional arousal between hunt and escape conditions.

Another possibility is that switching itself might have increased the emotional component or the level of arousal compared to staying. However, to our knowledge, none of the previous human studies of task switching showed an increased hypothalamic activation by the switching of the task including an “emotional” task switching ^2^ which task involves an emotional component and is more abstract than our task while not including a complex motor coordination as in our study. Finally, we tested whether the increased arousal affects the hypothalamic switching signal in the current study. We reasoned that if the hypothalamic switching is increased by the level of arousal, this signal should be stronger after the painful shock since it increases the arousal. To test this hypothesis, we performed a mixed-effect linear regression using the hypothalamic MVPSS as a dependent variable and the presence of electric shock before the switch as a fixed-effect variable. The result showed that shock right before the switch does not influence the hypothalamic MVPSS (t=0.92, p=0.354), showing that the hypothalamic switching signal is not influenced by the level of arousal. We also tested whether the hypothalamus-amygdala connectivity or Amygdala MVPSS is influenced by the pre-switch shock and results showed that neither of them was influenced by the pre-switch shock (all p>0.05). Therefore, considering that neither the hypothalamic switching signal nor its connectivity with the amygdala was associated with an increased arousal, it is likely that the hypothalamus is involved in the coordination of the switching between escape and hunt rather than simply encoding an arousal level.

**Effect of time on the performance and the hypothalamic switching signal**

Subjects completed the control condition after the Escape/Hunt task which raises a possibility that participants might have felt fatigue after finishing the first task which might have subsequently affected the performance of the control condition and decreased the hypothalamic BOLD signal. First, behaviorally, subjects showed fairly good performance (59% success) in the control condition meaning that they were engaged in the control condition. Second, we ran a mixed-effect regression using the hypothalamic MVPSS as a dependent variable and a trial number as a fixed-effect variable which showed that the hypothalamic switching signal does not decrease with time (t=-0.01, p=0.9990). Furthermore, in the mixed-effect logistic regression using the success in the trial as a dependent variable and a trial number as a fixed-effect variable, it turns out that the time did not affect the performance (t=1.45, p=0.2463). Based on these results, we concluded that the performance and hypothalamic switching signal in the control condition is less likely to be influenced by the order effect.

**References**

1. Satpute, A.B., Kragel, P.A., Barrett, L.F., Wager, T.D., and Bianciardi, M. (2019). Deconstructing arousal into wakeful, autonomic, and affective varieties. Neuroscience Letters *693*, 19–28. 10.1016/j.neulet.2018.01.042.

2. Reeck, C., and Egner, T. (2015). Emotional task management: neural correlates of switching between affective and non-affective task-sets. Social Cognitive and Affective Neuroscience *10*, 1045–1053. 10.1093/scan/nsu153.
